# Implementation and evaluation of the VA DPP clinical demonstration: protocol for a multi-site non-randomized hybrid effectiveness-implementation type III trial

**DOI:** 10.1186/s13012-015-0250-0

**Published:** 2015-05-12

**Authors:** Laura J Damschroder, Tannaz Moin, Santanu K Datta, Caitlin M Reardon, Nanette Steinle, Jane Weinreb, Charles J Billington, Matt L Maciejewski, William S Yancy, Maria Hughes, Fatima Makki, Caroline R Richardson

**Affiliations:** 1 Ann Arbor VA Center for Clinical Management Research, P.O. Box 130170, Ann Arbor, MI 48113-0170 USA; 2 VA Diabetes QUERI, Ann Arbor, MI USA; 3 VA Greater Los Angeles Healthcare System, Los Angeles, CA USA; 4 David Geffen School of Medicine, University of California, Los Angeles, CA USA; 5 Greater Los Angeles VA Health Services Research and Development (HSR & D) Center for Healthcare Innovation, Implementation and Policy, Los Angeles, CA USA; 6 Durham VA Medical Center, Durham, NC USA; 7 Duke University School of Medicine, Durham, NC USA; 8 Baltimore VA Medical Center, Baltimore, MD USA; 9 University of Maryland School of Medicine, Baltimore, MD USA; 10 Minneapolis VA Healthcare System, Minneapolis, MN USA; 11 University of Minnesota Medical Center, Minneapolis, MN USA; 12 Department of Family Medicine, University of Michigan, Ann Arbor, MI USA

**Keywords:** Diabetes prevention, Implementation, Veterans, Pragmatic study design

## Abstract

**Background:**

The Diabetes Prevention Program (DPP) study showed that lifestyle intervention resulted in a 58% reduction in incidence of type 2 diabetes among individuals with prediabetes. Additional large randomized controlled trials have confirmed these results, and long-term follow-up has shown sustained benefit 10–20 years after the interventions ended. Diabetes is a common and costly disease, especially among Veterans, and despite strong evidence supporting the feasibility of type 2 diabetes prevention, the DPP has not been widely implemented. The first aim of this study will evaluate implementation of the Veterans Affairs (VA) DPP in three VA medical centers. The second aim will assess weight and hemoglobin A1c (A1c) outcomes, and the third aim will determine the cost-effectiveness and budget impact of implementation of the VA DPP from a health system perspective.

**Methods/Design:**

This partnered multi-site non-randomized systematic assignment study will use a highly pragmatic hybrid effectiveness-implementation type III mixed methods study design. The implementation and administration of the VA DPP will be funded by clinical operations while the evaluation of the VA DPP will be funded by research grants. Seven hundred twenty eligible Veterans will be systematically assigned to the VA DPP clinical demonstration or the usual care VA MOVE!® weight management program. A multi-phase formative evaluation of the VA DPP implementation will be conducted. A theoretical program change model will be used to guide the implementation process and assess applicability and feasibility of the DPP for VA. The Consolidated Framework for Implementation Research (CFIR) will be used to guide qualitative data collection, analysis, and interpretation of barriers and facilitators to implementation. The RE-AIM framework will be used to assess Reach, Effectiveness, Adoption, Implementation, and Maintenance of the VA DPP. Twelve-month weight and A1c change will be evaluated for the VA DPP compared to the VA MOVE! program. Mediation analyses will be conducted to identify whether program design differences impact outcomes.

**Discussion:**

Findings from this pragmatic evaluation will be highly applicable to practitioners who are tasked with implementing the DPP in clinical settings. In addition, findings will determine the effectiveness and cost-effectiveness of the VA DPP in the Veteran population.

**Electronic supplementary material:**

The online version of this article (doi:10.1186/s13012-015-0250-0) contains supplementary material, which is available to authorized users.

## Background

### Preventing diabetes

The Diabetes Prevention Program (DPP) study showed that lifestyle intervention resulted in a 58% reduction in incidence of type 2 diabetes among individuals with prediabetes [1]. Additional large randomized controlled trials have confirmed these results [2-4], and long-term follow-up has shown sustained benefit 10–20 years after the interventions ended [3,5]. Table 1 lists published type 2 diabetes prevention clinical trials and their results. These trials provide a strong evidence base for large-scale dissemination and implementation of the DPP to prevent type 2 diabetes.

**Table 1:** **Published diabetes prevention program trial descriptions and selected outcomes**

Study	Individual or group	*N*(lifestyle group)	Years of follow-up	Mean weight loss (lifestyle group)	Reduction in developing diabetes
The Diabetes Prevention Program study [1]	Individual	1,079	2.8^a^	5.6 kg	58%
Diabetes Prevention Program Outcomes study [5]	Individual	910	10	2.0 kg	34%
The Finnish Diabetes Prevention Program study [42]	Individual	265	2	3.5 kg	58%^b^
China Da Qing Diabetes Prevention study [3]	Individual	266^c^	20	3.7 kg	43%
Indian Diabetes Prevention Programme [4]	Advice to individual^d^	133^e^	3	No significant change in body weight^f^	28.5%
DEPLOY YMCA [6]	Group	29	1	5.7 kg (6%)	NA
Group Lifestyle Balance [7]	Group	52^g^	1	5.5 kg (5.5%)	NA

^a^Follow-up ranged from 1.8–4.6 years.

^b^There was a 43% reduction in relative risk at a median of 7 years of follow-up; after a median of 4 years of active intervention period, participants who were still free of diabetes were further followed up for a median of 3 years [43].

^c^Number of participants, combined, across three lifestyle interventions: diet, exercise, and diet plus exercise.

^d^Participants received advice on healthy diet and regular physical activity.

^e^One hundred and thirty-three individuals were randomized to this intervention but 120 were available for follow-up at 2.5 years.

^f^Participants were younger (mean age 45.9 ± 5.7 years) and had lower BMI (25.8 ± 3.5 kg/m^2^) than previous studies.

^g^Number of participants that completed the 12-month assessment in a single group pre-post comparison study.

One significant barrier to implementing the DPP is the high cost of the individualized program. However, recent studies have shown that the program can be delivered at a lower cost in group sessions with comparable effectiveness [6,7]. For example, the Group Lifestyle Balance (GLB) program, a group-based adaptation of the DPP curriculum developed by the Diabetes Prevention Support Center (DPSC) of the University of Pittsburgh, resulted in clinically significant weight loss 12 months after baseline among program participants that completed the 12-month assessment [7,8]. GLB retains many of the components of the DPP, including standardized goals for weight loss, diet, and exercise and incremental introduction of self-regulation skills with a goal of self-regulation skill mastery.

Despite strong evidence supporting the feasibility of type 2 diabetes prevention, Veterans continue to be at high risk for developing type 2 diabetes. Primarily because of the older age of the population, the prevalence of type 2 diabetes is higher among Veterans who obtain care in the Veterans Affairs (VA) healthcare system than that in the general population, affecting approximately one in four Veterans [9]. In 2011, the Centers for Disease Control and Prevention (CDC) launched a national initiative to increase access to evidence-based DPP interventions for individuals with prediabetes [10], which contributed to the impetus for the current Veterans Affairs Diabetes Prevention Program (VA DPP) clinical demonstration.

### Preventing diabetes among Veterans

The VA National Center for Health Promotion and Disease Prevention (NCP) is the VA’s national program office responsible for many national disease prevention efforts. In 2006, NCP led the unprecedented implementation of VA MOVE!®, a national weight loss program within the VA [11]. VA MOVE! is an evidence-based, multi-disciplinary, comprehensive weight management program [11-14]. The core program includes eight to ten face-to-face small group sessions, led by a multi-disciplinary team of nutritionists, health psychologists, and physical therapists. While many elements of VA MOVE! are comparable to the VA DPP, Table 2 lists program design differences between the VA DPP and VA MOVE!. Additional file 1 provides further details. Within VA, over 95% of Veterans are screened for obesity, provided with obesity counseling, and offered VA MOVE! or another weight management program, as clinically appropriate [15,16]. Veterans are candidates for VA MOVE! if their body mass index (BMI) is greater than 30 kg/m2 or greater than 25 kg/m2 with one obesity-related condition. However, Veterans are not routinely screened for prediabetes, and with only a few exceptions, lifestyle modification programs that specifically target individuals with prediabetes are not available in the VA.

**Table 2:** **Program design differences between the VA DPP and VA MOVE!**

Program attribute	VA DPP	VA MOVE!	Behavioral constructs impacted
Goal awareness and commitment	Assigned generic goals	Patients create own goals	Goal awareness
			Goal commitment
Outcome expectations	Focus on diabetes prevention	Focus on weight loss and healthy lifestyle	Perceived risk [44]
			Outcome expectations [45]
			Intrinsic/extrinsic motivation [46]
Group cohesion	Closed cohorts, single coach, group identity around prediabetes	Open and closed cohorts, multi-disciplinary team, individuals with diabetes, prediabetes and normal glucose tolerance in the same group	Group cohesion
			Attitude toward others in group
			Attitude toward the group coach
Self-regulation skill mastery	Iterative skill building and a focus on mastery	Independent sessions addressing a series of behavioral skills	Self-regulation skills [47]
			Self-efficacy [47,48]
			Willingness to self-monitor
			Adherence to monitoring
Program intensity	Sixteen sessions in 6 months	Twelve to fourteen sessions in 3 months	Attendance
			Satisfaction with number of visits
			Maintenance

Executive leaders identified the implementation of the DPP in the VA as a top-priority clinical goal and gave NCP the lead for the current initiative. In order to evaluate the feasibility of implementing a group-based DPP in the VA, NCP will conduct a clinical demonstration of the DPP at three VA medical centers across the country, hereafter referred to as the VA DPP. NCP requested the assistance of the research coordinating center to conduct a comprehensive and rigorous evaluation of the implementation of the VA DPP in preparation for potential national dissemination as well an evaluation to determine effectiveness of the VA DPP in the Veteran population. Each VA DPP site will screen and enroll Veterans with prediabetes who have been referred to the VA MOVE! program. A systematic assignment algorithm will assign these Veterans either to the VA DPP or the VA MOVE! program.

The VA DPP has been deemed a clinical quality improvement (QI) initiative. The implementation and administration of the VA DPP will be funded by clinical operations while the evaluation of the VA DPP will be funded by research grants. Because of the extremely short timeline, the research coordinating center submitted two separate research proposals, which are detailed in this protocol. Each research proposal was reviewed by a national peer review panel. The first proposal was submitted as part of the Quality Enhancement Research Initiative (QUERI) Rapid Response Project (RRP) program, which funds 1-year studies. This grant will enable the research coordinating center to collect baseline data as the VA DPP begins. The research coordinating center then submitted a QUERI Service Directed Project (SDP), which funds 2-year studies. This grant will enable the research coordinating center to collect and analyze follow-up data. Table 3 details the different funding sources and institutional review board (IRB) approvals for evaluation activities.

**Table 3:** **VA DPP funding sources and IRB approvals**

Aims	Data type	Funding	IRB approvals
Quantitative data			
2	Weight and A1c lab results: baseline and follow-up	NCP QI^a^	Data collection approved as QI
		SDP^b^	Data access and analysis approved by five IRBs^c^
2, 3	Baseline patient surveys	RRP^d^	Approved by five IRBs
2, 3	Follow-up patient surveys	SDP	Approved by five IRBs
3	EQ5D survey	RRP	Approved by five IRBs
		SDP	
2, 3	Administrative utilization data linked to patient survey data	SDP	Approved by five IRBs for consented patients only
3	Staff time logs	NCP QI	Data collection approved as QI
			Data access and analysis approved by the research coordinating center’s IRB and the site conducting the cost analyses IRB
			• Included as information only in IRB applications for sites implementing the VA DPP
Qualitative data			
1	Early implementation staff interviews	NCP QI	Data collection approved as QI
		SDP	Data access and analysis approved by the research coordinating center’s IRB
			• Included as information only in IRB applications for sites implementing the VA DPP
1	Late implementation staff interviews, site visit field notes	SDP	Approved by the research coordinating center’s IRB
			• Included as information only in IRB applications for sites implementing the VA DPP
2	Patient interviews	SDP	Approved by the research coordinating center’s IRB
			• Included as information only in IRB applications for sites implementing the VA DPP
1	Fidelity assessments, meeting notes, document artifacts (e.g., emails)	NCP QI	Data collection approved as QI
		SDP	Data access and analysis approved by the research coordinating center’s IRB
			• Included as information only in IRB applications for sites implementing the VA DPP

^a^QI: quality initiative funded by the National Center for Health Promotion and Disease Prevention (NCP).

^b^SDP: Service Directed Project funded by the VA Quality Enhancement Research Initiative (QUERI) program; 2 year grant (12–549).

^c^IRB: Institutional Review Boards are located at the three sites implementing the VA DPP, the research coordinating center, and the site conducting the cost analyses.

^d^RRP: Rapid Response Project funded by the VA QUERI program; 1 year grant (12–440).

### VA DPP design

The VA DPP will run multiple cohorts per site, with the time intervals between the start of each cohort to be determined by site capacity. Each VA DPP cohort will include a series of 22 face-to-face GLB sessions using GLB materials, led by a coach with formal nutrition training and prior lifestyle coaching experience [17]. Table 4 details eligibility criteria for the VA DPP. Details about sample size and recruitment are provided under the “Aim 2” section below.

**Table 4:** **VA DPP eligibility criteria**

Inclusion criteria	Exclusion criteria
Patients will be considered VA DPP eligible if all of the below are true:	Patients will be considered VA DPP ineligible if one or more of the following is true:
Patient has fasting glucose of 100–125 mg/dl OR A1c of 5.7%–6.4% for the past 6 months.	Patient already has type 2 diabetes. If patient was ever diagnosed with diabetes, they are not eligible, even if their A1c level is within the eligible range; they are then considered “diet controlled”.
Note: This is NOT the same as the VA’s criteria, which considers prediabetes as 5.7%–6.9%.	
	Patient does not have laboratory-confirmed elevations in either fasting glucose or A1c that indicates prediabetes.
Patient is VA MOVE! eligible (BMI ≥ 30 or BMI ≥ 25 with at least one cardiovascular disease risk factor).	
	Patient is taking metformin or other hypoglycemic agent.
	Patient is currently known to be pregnant.
Patient is competent to provide informed consent.	
	Patient has an eating disorder.
Patient understands English.	Patient has a medical contraindication to diet, exercise, or weight loss.
Patient has not used metformin for the past 3 months.	Patient is not competent to complete the informed consent process.
Travel time is <60 min from the VA medical center where patient receives their care.	Patient has attended all or part of VA MOVE! in the last year.
Patient has completed VA MOVE! introduction class.	Travel time is >60 min from the VA medical center where patient receives their care.

### VA DPP implementation strategy

**Figure 1:**
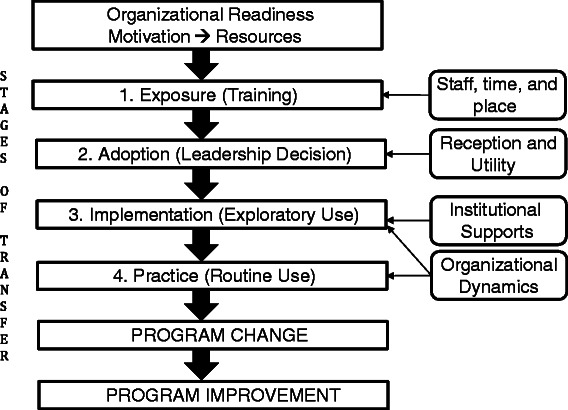
VA DPP strategic framework for implementation.

The strategic framework for implementing the VA DPP will be an adaptation of Simpson et al.’s theoretical program change model for translating research into practice [18]. Figure 1 illustrates components of this framework.

Organizational readiness to implement a new program is demonstrated by stakeholders being internally motivated or externally pressured for change, which results in the allocation of resources necessary for implementation and administration of a new program [18]. The VA DPP site leaders must show commitment to diabetes prevention in order to successfully implement the VA DPP. NCP will provide all three VA DPP sites with program resources, including funding for staffing, training, and program materials.

### Phase one: adoption

In the VA DPP, exposure and training will take place after each VA DPP site’s Medical Center Director signs a formal memorandum of understanding (MOU) with NCP to demonstrate their commitment to the program. A clinical champion at each site will be responsible for shepherding the MOU through the approval process. This will require a series of presentations, conference calls, and meetings to inform all levels of local leadership about the VA DPP and to obtain a formal commitment from senior leaders to visibly support the project. The clinical champion will be responsible for hiring staff and coordinating with other site-specific resource teams that will be needed for the VA DPP, including the laboratory and the medical records teams. NCP leadership will help identify local supporters and provide visible authority (e.g., phone calls to leaders) at each site to further convey the importance of the project as it is being implemented.

#### Phase two: training

All team members will be trained at a 2-day GLB training program offered by the DPSC at the University of Pittsburgh. NCP will pay the costs for round-trip travel and registration for the training.

#### Phase three: implementation (exploratory use)

VA DPP cohorts will run in parallel to existing VA MOVE! groups. Implementation will rely on the existing VA MOVE! infrastructure for obesity screening, program referral, and medical clearance. Each site will develop a site-specific protocol that aligns the new processes for screening, identifying, informing, and assigning Veterans to the VA DPP within the existing VA MOVE! infrastructure. Protocols will also include processes for using the electronic medical record (EMR) system to track Veterans with prediabetes by VA DPP cohort. These institutional supports will be essential for monitoring and providing feedback to ensure successful implementation and administration of the VA DPP. The research coordinating center will facilitate weekly calls with VA DPP staff from each site to get status updates and provide collective problem-solving support during the implementation process.

#### Phase four: sustainability (routine use)

If the VA DPP is found feasible and effective, the challenge will be to sustain the program at each site and disseminate it more broadly within the VA. The goal of the evaluation study is to provide recommendations to NCP about how to scale-up the VA DPP, or its more successful components, at a national level.

### Research study aims

The research study design will follow the structure of an effectiveness-implementation hybrid type III trial [19]. Because of the strong evidence base for the DPP, the primary aim of this study will facilitate and evaluate implementation of the VA DPP at the three sites. However, because the DPP has not yet been evaluated in a Veteran population, the second aim will assess weight and hemoglobin A1c (A1c) outcomes for Veterans who enroll in the VA DPP. The third aim will determine the cost-effectiveness and budget impact of implementation of the VA DPP from a health system perspective. Specifically, the research study aims are:

#### Aim 1

The first aim is to evaluate an implementation strategy and identify barriers and facilitators to implementation of the VA DPP at three VA medical centers.

#### Aim 2

The second aim is to evaluate the effect of program implementation on participant outcomes, specifically weight and A1c values at 12 months, in the VA DPP compared to VA MOVE!, and assess patient engagement and adherence in the VA DPP.

#### Aim 3

The third aim is to conduct cost-effectiveness and budget impact analyses of implementing the VA DPP in the VA from the health system perspective.

## Methods

In this section, the VA DPP evaluation aims, study participants, data collection procedures, and analysis plans will be described for each study aim.

### Aim 1: implementation evaluation

This study will not be sufficiently powered to quantitatively assess site-specific factors that may influence implementation outcomes. Therefore, this study will conduct a mixed methods, multi-dimensional, formative evaluation of the implementation strategy. This formative evaluation will incorporate the following features: 1) developmental evaluation to identify barriers and facilitators to implementation; 2) implementation and progress-focused evaluation to actively monitor implementation progress and adapt the program as needed; and 3) interpretive evaluation to provide detailed information on success or failure of implementation [20]. If the program is effective in the Veteran population, findings will be used to develop recommendations for disseminating the VA DPP more broadly in the VA.

#### Evaluation framework

**Figure 2:**
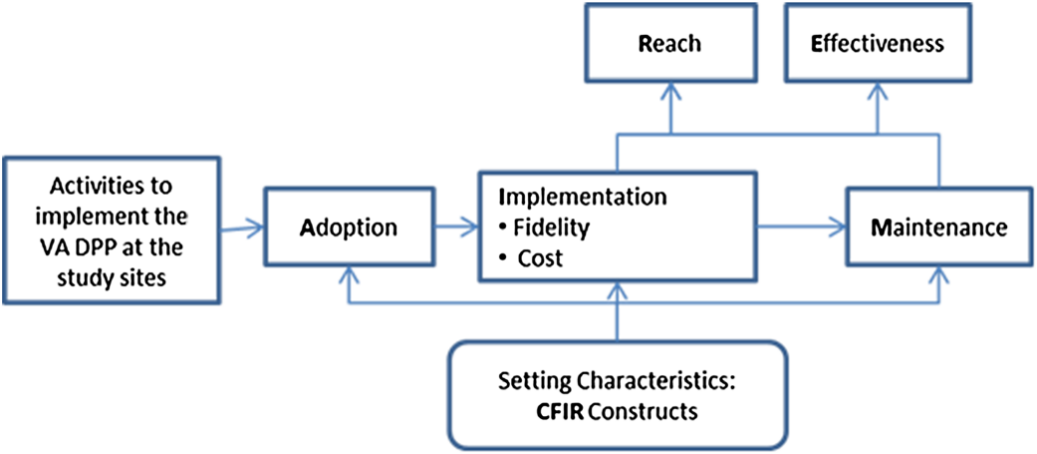
Formative evaluation framework: integration of RE-AIM and the CFIR.

This study will integrate two published frameworks to guide the formative evaluation: 1) the RE-AIM framework [21] will be used to assess external validity and prospects for dissemination and implementation of the VA DPP in the VA and 2) the Consolidated Framework for Implementation Research (CFIR) will be used to systematically assess contextual factors that influence adoption and implementation [22]. Figure 2 illustrates the integration of RE-AIM and CFIR. Additional file 2 provides detailed research questions and quantitative and qualitative measures for each of the RE-AIM dimensions.

The CFIR will be used to guide a systematic evaluation of barriers and facilitators that may affect adoption, implementation, and/or maintenance. The CFIR comprises 39 common constructs from across published implementation frameworks and models that are believed to influence implementation [23-25]. These constructs are organized within five major domains: intervention characteristics, outer and inner setting, characteristics of individuals, and implementation process. Examples of constructs include the relative advantage of the program (intervention characteristics), patient needs and resources (outer setting), networks and communications in the facility (inner setting), knowledge and beliefs of stakeholders (characteristics of individuals), and engaging stakeholders in the process (process).

#### Study participants

Participants will include VA DPP staff, VA MOVE! program coordinators and staff, Health Promotion and Disease Prevention (HPDP) program managers, Health Behavior Coordinators (HBC), primary care clinical leaders, primary care providers, and other primary care staff at each VA DPP site.

#### Data collection

##### Staff interviews

The CFIR will be used to develop a semi-structured interview guide and guide data collection. Additional file 3 provides copies of the staff interview guides. Stakeholders (four to ten per site) will be interviewed at early (or pre-) implementation and late (or post-) implementation of the VA DPP. Interviews will take place over the phone or in person during site visits and will last 15–60 min. The early implementation interviews will assess receptivity to the VA DPP and identify potential implementation barriers and facilitators. This information will be used to develop tailored implementation and communication strategies for each site. The late implementation interviews will elicit in-depth information about experiences implementing and administering the VA DPP and to understand success or failure of implementation. These interviews will explore prospects for sustainability and inform broader dissemination and implementation if the program is effective. In addition, field notes, project meeting notes, and other artifacts (e.g., emails) will be used throughout the course of the study in order to identify and address barriers to implementation, refine the program, and provide feedback to site staff.

##### Fidelity assessments

Fidelity checklists will be completed for two VA DPP and two VA MOVE! sessions in each cohort. The project coordinator will use the checklist to rate each item and provide open-ended notes. This approach will provide fidelity assessments of VA DPP delivery and a comparison to VA MOVE! sessions. Additional file 4 provides copies of the fidelity checklists.

#### Data analysis

##### Staff interviews

The CFIR will be used as an organizing framework for qualitative data coding and analysis. Deductive and inductive content analyses, guided by a consensus-based qualitative research approach [26,27], will be conducted on all interview data using NVivo qualitative analysis software. This analysis approach will have the following features: 1) multiple judges will be used throughout data analysis to foster multiple perspectives; 2) consensual validation will be achieved through a process of discussion and deliberation [28]; 3) an expert auditor not integrally involved in the study will review the process to help maximize validity of findings; and 4) CFIR constructs will be identified and applied to cases, and cross-analyses will be performed [29]. A summary will be written for each facility based on data from verbatim interview transcripts and field notes with supporting quotes. Matrices will be constructed to help discern patterns within and across facilities by comparing and contrasting themes. Qualitative analysts will be blinded to facility outcomes related to implementation effectiveness until qualitative data analyses are completed. Full information from all facilities will be used in the interpretative phase, when qualitative findings will be combined with quantitative data measuring implementation effectiveness.

##### Fidelity assessments

Fidelity ratings will be used to compute average scores for coaching and delivery fidelity domains. Simple *t*-test analyses will be used to assess differences between the VA DPP and VA MOVE!.

### Aim 2: effectiveness evaluation

This study will evaluate whether patient weight and A1c outcomes are better in the VA DPP than those in VA MOVE!. In addition, process measures will be assessed, including the percentage of Veterans who engage in (attend at least four sessions) and adhere to (attend at least nine sessions) the VA DPP [30]. Careful attention will be paid to discern whether there are significant differences across important demographic sub-groups, such as gender or race/ethnicity in program outcomes (weight and A1c outcomes) or process measures (enrollment, engagement, and adherence). Mediation analyses will also be conducted to identify whether program design differences between the VA DPP and VA MOVE! impact outcomes.

#### Study participants

Participants will include eligible Veterans who are referred to VA MOVE! at each of the three VA DPP sites. Table 4 details eligibility criteria. Power analyses were conducted to determine sample size needed to have 80% power to detect expected differences in program outcomes with alpha of .05, assuming two-tailed testing. The power analysis assumed that the VA DPP would result in 1.7 additional pounds of weight loss compared to VA MOVE! with a standard deviation of 6.96 lbs (assuming a standard deviation of 11 lbs and a within-person correlation of .8 for weights at baseline versus 12 months) and assuming an intra-group correlation coefficient of .014. Assuming that 20% of participants would drop out by 12 months, this calculation indicated the need to recruit 720 patients (360 for the VA DPP and 360 for VA MOVE!). Given the sample size, the expected pace of recruitment, a target of 20 participants per cohort, and the structure of the existing VA MOVE! programs, six cohorts will be conducted with a new cohort starting every 2 months. Since randomization is not possible for this clinical demonstration, a systematic assignment process will be used; every 2 months, the first 20 patients who meet the VA DPP eligibility criteria will be enrolled and assigned to the VA DPP and the next 20 eligible patients will be assigned to VA MOVE! by VA DPP site staff.

#### Data collection

##### Patient clinical outcomes

Weight will be measured with a medical-grade scale at each VA DPP and VA MOVE! session as well as at a 12-month program-specific session. Weight will also be routinely measured and documented in the EMR during other VA clinical visits. Baseline A1c and 12-month follow-up A1cs will be ordered by providers or the VA DPP coaches. A1c testing will occur via a laboratory blood draw or point of care (POC) testing. Attendance at all VA DPP and VA MOVE! sessions will be documented in the EMR by program session leaders.

#### Patient surveys

Eligible Veterans will be invited to complete baseline surveys after completing an informed consent process. Consented Veterans will be given a paper copy of the survey along with a business reply envelope addressed to the research coordinating center. At 12 months, Veterans will be invited to complete follow-up surveys. Survey instructions will include a web address that will link to an electronic version of the paper survey for Veterans who prefer to answer the survey online. The surveys will measure potential mediators of program outcomes, including perceptions of goal awareness and commitment, outcome expectations, group cohesion, self-regulation skill mastery, and program intensity, as well as quality of life, satisfaction with the program, sleep, social support, and depression. Additional file 5 provides a copy of the program surveys.

#### Patient interviews

At 12 months, patients will be purposively selected for interviews in order to maximize variation in program arm, participation, weight loss, and sex. Table 5 illustrates the sampling strategy. Interviews will assess patient experiences in the program. Additional file 6 provides a copy of the patient interview guide.

**Table 5:** **Sampling strategy for patient semi-structured interviews**

Axis of diversity	Strata
Program	• VA DPP
	• VA MOVE!
Level of participation	• High participation
	• Low participation
	• No participation
Category of weight loss	• High weight loss
	• Low weight loss, maintenance, or weight gain
Sex	• Women
	• Men

#### Data analysis

##### Patient surveys and clinical outcomes

Effects of the VA DPP and VA MOVE! on patient outcomes at 12 months will be assessed using clinical weights and A1c lab values. The distribution of all study variables and baseline differences in factors that may affect study outcomes between groups will be assessed. If significant differences are found, they will be included as covariates in the final analyses. At each assessment time, cross-sectional means and their associated 95% confidence intervals for continuous outcomes (e.g., weight) will be described. Comparison between the VA DPP and VA MOVE! in weight loss at 12 months will be completed with a linear mixed-effects model, using program arm as random intercepts to account for potential intra-program group correlation. The model will include the primary predictor of program arm (the VA DPP or VA MOVE!), indicators for the three study sites, baseline weight values, and age, sex, and race variables. Time-averaged effects of the VA DPP over VA MOVE! will be compared using weight loss at 12 months as the dependent variable. The model will allow the use of all available data, even if some Veterans are missing 12-month weights, and will give an unbiased estimate as long as missingness does not depend on the unobserved missing outcomes. Careful graphical analyses of longitudinal outcome data will be completed to ensure that an appropriate and meaningful longitudinal model is created. Additional file 7 describes the planned mediation analyses.

##### Patient interviews

Inductive content analysis, guided by a consensus-based qualitative research approach, will be conducted on all interview data [26,27] using NVivo qualitative analysis software.

### Aim 3: economic evaluation

Cost-effectiveness of a program and its net impact on healthcare budget are important factors for decision makers that are considering implementing new programs. Therefore, an economic analysis of the VA DPP will be conducted to inform decision makers about implementation of the VA DPP in the VA. A combination of approaches in the economic analysis will be used. A direct measurement micro-costing approach will be used to assess program-related costs. In addition, modeling will estimate long-term resource-utilization costs and health outcomes. Together, this will estimate the incremental cost-effectiveness of the VA DPP [31] and create a complete picture of VA expenditures and health events over time among Veterans with prediabetes. The cost results from this VA study population will then be used to extrapolate the overall budget impact if the VA DPP is implemented for all Veterans with prediabetes in the VA healthcare system.

#### Evaluation framework

The recommendations of the Panel on Cost-effectiveness in Health and Medicine appointed by the US Public Health Service on the Reference Case approach will guide the cost and cost-effectiveness analyses [32]. The Reference Case approach requires that the cost-effectiveness analysis be conducted from the societal perspective and use a time horizon long enough to adequately capture important future benefits, harms, and costs that occur due to program implementation, where health outcomes and resource utilization changes of all relevant stakeholders are taken into consideration. The Reference Case approach dictates that morbidity and mortality be reflected in a single measure, the quality-adjusted life year (QALY), and that future costs and QALYs be discounted to present value.

The principles of good practice for budget impact analysis outlined by the International Society of Pharmacoeconomics and Outcomes Research (ISPOR) Task Force will be followed in the budget impact analysis [33], as done in previous VA studies [34,35]. The budget analysis will primarily focus on short-term costs and will take into consideration resource constraints and limitations in the health system or in the patient population that may limit the proportion of the target population served.

#### Study participants

Participants will include VA DPP staff as well as patients in the VA DPP at each VA DPP site.

#### Data collection

##### Costs

Micro-costing largely involves direct measurement of the labor inputs required to conduct the VA DPP and VA MOVE! sessions. Labor cost includes the fixed cost of training the VA DPP coaches, as well as the variable cost of delivering the VA DPP to each Veteran. Additional file 8 provides a copy of the instructions and log sheet that will be used by VA DPP staff to log their activity time. The training and clinical demonstration-related time for each staff member will be multiplied by his or her respective wage rate (including fringe benefits), aggregated, and then divided by the number of patients in the VA DPP to derive per-person labor cost. Equipment needed to deliver the VA DPP includes patient workbooks and log books, as well as token patient incentives that are unrelated to research survey completion, including an inexpensive kitchen scale and water bottle.

For program cost of patients in the VA MOVE! comparison group, the costs of VA MOVE!-specific visits from the VA’s Managerial Cost Accounting System (MCAS) outpatient extract file will be collected; VA MOVE! visits can be directly identified by a clinic identifier in VA claims.

The VA healthcare system also incurs substantial indirect costs, such as administrative costs, janitorial services, and utilities, which are not specific to a health service. An indirect cost multiplier using the total indirect and direct cost variables in the VA’s MCAS extract file will be calculated and applied to the above direct cost estimates to derive total (direct + indirect) cost. Health-care resource utilization and costs incurred by patients during the period they were enrolled in the program will be extracted from the MCAS outpatient, inpatient, and pharmacy extract files located in the VA’s Corporate Data Warehouse (CDW).

##### Effectiveness

Following the recommendations of the Panel on Cost-effectiveness in Health and Medicine, QALYs will be used as the effectiveness measure [32]. Utility values will be measured using the EuroQOL EQ-5D-5 L (EQ5D) in baseline and follow-up program surveys to calculate QALYs during the study period [36]. Additional file 5 provides a copy of the EQ5D survey. The EQ5D asks patients to rate their quality-of-life for the following five dimensions: mobility, self-care, usual activities, pain or discomfort, and anxiety or depression. There are five statements for each domain describing increasing levels of disability; patients are asked to choose the statement that best represents their quality-of-life for that dimension. Based on previous research by the EuroQoL group, country-specific valuation sets have been derived from the general population that allow the responses elicited by the EQ5D to be converted into utility values [37]. The US EQ5D value set will be used in the analysis.

##### Analysis

Data analysis will begin with standard descriptive statistics, univariate analysis, and bivariate comparisons of the VA DPP and VA MOVE! session costs and the utility values derived by the EQ5D. A lifetime timeframe for the cost-effectiveness analysis will be adopted because the impact of the VA DPP is not likely to manifest until many years in the future. The incremental cost-effectiveness ratio (ICER) will be calculated as the difference in the cost per Veteran between the VA DPP and VA MOVE! groups, divided by the difference in the QALYs per Veteran in both groups. As recommended by the Panel on Cost-effectiveness in Health and Medicine, future costs will be deflated to present value and costs and QALYs will be discounted by 3% to represent current year equivalency [32].

The CDC-RTI Diabetes Cost-Effectiveness Markov model (CDC-RTI model) will be used to estimate the ICER of the VA DPP and VA MOVE!. The CDC-RTI model is a well-validated model for estimating lifetime costs and outcomes associated with diabetes and its concomitant comorbidities [38]. The CDC-RTI model is a Markov simulation model that estimates disease progression and cost-effectiveness for type 2 diabetes and was used to assess cost-effectiveness in the original DPP study [38]. In the CDC-RTI model, progression between disease health states is dictated by the transition probabilities that depend on risk factors including glycemic control (measured by A1c), duration of diabetes, and blood pressure, as well as other patient and clinical characteristics. The VA DPP and VA MOVE! group will differentially affect these transition probabilities and resulting complications. Initial inputs in the CDC-RTI model will include patient characteristics, changes in A1c and blood pressure, program, and healthcare utilization costs, and the utility values discussed above. The model will then simulate development of common complications of diabetes, such as nephropathy, neuropathy, retinopathy, coronary heart disease, and stroke. CDC-RTI model outcomes will include complications, deaths, costs, QALYs, and the ICER. Monte Carlo simulation of the model will allow for consideration of a range of model parameter values. This will generate a 95% confidence interval around the ICER point estimate. In addition, a series of one-way sensitivity analyses to assess the robustness of the results will be conducted to identify which model parameters are most sensitive to changes and thus driving the results.

The budget impact analysis will use epidemiologic evidence from the literature [31,39] and projections provided by the VA Office of Policy and Planning [40,41] to estimate the proportion of the Veteran population that receives their care from the VA healthcare system who are likely to have prediabetes. These analyses must also factor in real-world complications of program implementation, such as resource constraints or potential lack of Veteran engagement or adherence to the VA DPP. Therefore, a sensitivity analysis will be included to estimate the budget impacts of different proportions of the Veteran population with prediabetes being serviced by the VA DPP.

## Discussion

This research study will be unique in its innovative partnership and pragmatic evaluation design, which includes three aims: evaluating implementation, assessing weight and A1c outcomes, and determining the cost-effectiveness and budget impact of implementation and administration of the VA DPP in the VA. Design trade-offs were assessed and balanced to meet the need for both external and internal validity; the study will determine if it is feasible to implement the VA DPP on a large scale and if the VA DPP achieves expected outcomes for Veterans. The interplay between the VA DPP implementation and administration (the clinical quality improvement component) and the VA DPP evaluation (the research component) will present unique benefits as well as challenges.

Because the DPP has a strong evidence base, it is appropriate to prioritize maximization of external validity. Due to the infeasibility of obtaining individual patient consent and randomization in a clinical setting, assessment of the VA DPP weight outcomes will rely on a population-based non-randomized study design. This design was chosen because it provides an acceptable degree of internal validity given pragmatic constraints. The evaluation will determine the infrastructure needed to implement and administer the VA DPP, especially within the context of the existing VA MOVE! program. This approach will enable the use of existing clinical screening and referral processes for VA MOVE!. Attention to external validity considerations will result in a sustainable program that the VA can run on a national scale, which may mean building on existing infrastructure, rather than focusing on the VA DPP in isolation.

The research coordinating center’s close working partnership with NCP will enable them to leverage funding from multiple sources. NCP will provide a clinical operations budget to fund the VA DPP implementation and administration. The research coordinating center will augment this funding with research grant money to fund the VA DPP evaluation. The partner- and implementation-focused research funding program in VA promotes this kind of work.

However, there will be regulatory challenges. Five separate IRB approvals from the three VA DPP sites, the research coordinating center, and the site conducting the cost analyses will be required. The time needed to obtain these approvals will be significant. NCP will be actively implementing the VA DPP and starting to recruit the first patients, and it will be imperative that necessary approvals are in place before the first baseline survey can be administered. The tension between the two timeframes will be especially challenging to manage in the early phases of the VA DPP implementation. It will be important to devote considerable time for education and dialogue with IRB administrators to negotiate boundaries between clinical QI data and research data. In addition, study protocols need to be established to enable access to the clinical QI data for research purposes as needed. This confusion may extend to VA DPP staff as well; frequent and ongoing discussions will occur to elucidate whether VA DPP staff members will be acting with their clinical or research “hat” on. For example, patient assessment for eligibility for the VA DPP will be considered a clinical activity while administering a baseline survey to patients will be considered research.

The use of multiple data sources will provide a detailed understanding how the VA DPP should be implemented nationally as well as the effectiveness of the VA DPP. The use of the CFIR and RE-AIM frameworks to guide data collection and analysis for the formative evaluation will provide an efficient approach for assessing and reporting findings to NCP and the VA DPP sites. Deductive and inductive analysis ensures consideration of themes or findings outside of these frameworks, which will help bolster validity of findings. Collecting information about fidelity and characteristics of program delivery will allow for comparison of the programs, as well as assessment of potential association with weight and A1c outcomes. The combination of qualitative and quantitative data elicited from patients will reveal greater insights about patient experiences with both programs.

## Conclusion

This study is unique in highlighting the tremendous rewards and unique challenges of conducting research in close partnership with clinical leaders. Findings from this pragmatic evaluation will be highly applicable to practitioners who are tasked with implementing the DPP in clinical settings. In addition, findings will determine the effectiveness and cost-effectiveness of the VA DPP in the Veteran population.

### Trial status

Trial status at the time of manuscript submission is ongoing. Patients have been recruited, and baseline assessments are complete. Data cleaning or analysis of 12-month summative outcomes has not been started.

## Additional files

Additional file 1: **VA DPP and VA MOVE! program design differences.** This file provides an outline of how the VA DPP and VA MOVE! differ. Additional file 2: **Research questions and RE-AIM measures.** This file provides a detailed list of research questions and quantitative and qualitative measures for RE-AIM domains. Additional file 3: **Staff interview guides.** This file provides a copy of the master interview guides (early and late implementation) used to interview staff. Additional file 4: **Session delivery fidelity checklists for the VA DPP and VA MOVE!.** This file includes copies of fidelity checklists used to assess VA DPP fidelity to the GLB program and to compare VA DPP sessions to VA MOVE! sessions. Additional file 5: **Program and EuroQOL EQ-5D-5 L surveys.** This file provides a copy of the program and EuroQOL EQ-5D-5 L surveys. Additional file 6: **Patient interview guide.** This file provides a copy of the master interview guide used to interview patients. Additional file 7: **Mediation analyses.** This file describes the mediation analyses in the “Aim 2” section. Additional file 8: **Cost-effectiveness assessment instructions and log.** This file provides a description and instructions for staff to use to log their time.

## Abbreviations

A1c: Hemoglobin A1c
BMI: Body mass index
CDC: Centers for Disease Control and Prevention
CDC-RTI: CDC-RTI Diabetes Cost-effectiveness Markov model
CDW: Corporate Data Warehouse
CFIR: Consolidated Framework for Implementation Research
DPP: Diabetes Prevention Program
DPSC: Diabetes Prevention Support Center
EMR: Electronic medical record
EQ5D: EuroQOL EQ-5D-5L
GLB: Group Lifestyle Balance
ICER: Incremental cost-effectiveness ratio
IRB: Institutional review board
ISPOR: International Society of Pharmacoeconomics and Outcomes Research
MCAS: Managerial cost accounting system
MOU: Memorandum of understanding
NCP: National Center for Health Promotion and Disease prevention
POC: Point of care
QALY: Quality-adjusted life year
QI: Quality improvement
QUERI: Quality Enhancement Research Initiative
RRP: Rapid Response project
SDP: Service Directed Project
VA: Veterans Affairs
