# Supplementary material for: Implementation and evaluation of the VA DPP clinical demonstration: protocol for a multi-site non-randomized hybrid effectiveness-implementation type III trial
Source: Implement Sci. 2015 May 12;10:68. doi: 10.1186/s13012-015-0250-0 (PMC4429938; doi:10.1186/s13012-015-0250-0)
Supplement: Additional file 1: — VA DPP and VA MOVE! program design differences. This file provides an outline of how the VA DPP and VA MOVE! differ. [file 13012_2015_250_MOESM1_ESM.pdf]

VA DPP Study Protocol Additional File 1: VA DPP and VA MOVE! Program Design Differences

VA MOVE! is a lifestyle change weight loss program that incorporates some but not all of the core components of the original DPP. While VA MOVE! and the VA DPP are similar in many ways, there are a few critical differences between the two programs.

Goal Awareness and Commitment: In VA MOVE!, participants learn about characteristics of SMART (specific, measurable, attainable, relevant, and timely) goals and are encouraged to set their own goals with respect to diet, exercise, and weight loss. In contrast, the VA DPP assigns relatively generic fixed goals (7% weight loss in six months, 150 minutes of moderate intensity exercise per week, and < 25% of calories from fat) for all participants. These different approaches to goal setting have the potential to impact motivation, effort to meet goals, and successful goal attainment. The Theory of Self-Determination [1] makes the distinction between intrinsic and extrinsic motivation and between autonomous and controlled regulation. Intrinsically motivated goals are set by the individual, and achieving the goal is experienced as a reward in and of itself. In contrast, extrinsically motivated goals are imposed by another person, and achieving the goal results in an extrinsic reward accruing to the individual, such as a prize or a good grade, but will not be experienced as rewarding in and of itself.

According to the Theory of Self-Determination, intrinsic motivation and autonomous regulation are more likely to result in successful goal attainment than extrinsic motivation and controlled regulation. However, empirical results from recent studies have called into question the assumption that intrinsically self-set goals are more effective motivators than extrinsically imposed goals. In a randomized controlled trial by Resnicow et al. comparing autonomy support to directive health communication about fruit and vegetable consumption, the majority of participants at baseline expressed support for the statement: “In general, when it comes to my health I would rather an expert just tell me what I should do” [2]. Furthermore, the group assigned to the autonomy supportive intervention did not do better than those assigned to the directive intervention [2]. Another recent study designed to demonstrate that non-directive e-coaching would be more effective than directive e-coaching actually showed that directive e-coaching resulted in greater weight loss [3]. Other investigators have speculated that autonomy supportive interventions for weight loss may be more important for women than for men [4-6]. Veterans tend to be male and have been trained to take orders. As such, it is hypothesized that the directive goals in the VA DPP will result in more weight loss than the autonomous self-set goals in VA MOVE!.

Outcome Expectations: The VA DPP focuses on diabetes prevention for patients at increased risk of developing diabetes while VA MOVE! focuses on weight loss and a healthy lifestyle for a broad range of patients with obesity or overweight and associated risk factors. It is possible that these goals have a different impact on goal striving and goal attainment. Specifically targeting individuals at high risk for developing diabetes may alter critical behavioral constructs such as outcome expectations and perceived risk. In addition, a group of individuals at a similar disease stage (prediabetes) may be able to provide more effective social support than those in a group at widely varying stages of disease.

Group Cohesion: Because the VA MOVE! program at most VA medical centers is designed to allow Veterans some flexibility in which sessions they attend, groups are not completely closed. For example, in most VA MOVE! programs, Veterans who miss a session can make up that session by sitting in on

another group's session that covers the topic they missed. Additionally, the session leader is not the same from session to session. For example, a physical therapist may lead the physical activity session while a dietitian may lead the session on calories and macronutrients. The lack of continuity in the groups may help Veterans because of its flexibility, but it may compromise group identity formation, which might in turn lead to decreased commitment to the group. In the VA DPP, a single coach will be leading all of the sessions in a particular cohort. In addition, the cohort will be closed, with no new members participating in subsequent sessions. It is hypothesized that this will increase group identity and group cohesion, and that this will in turn increase participation rates in the VA DPP.

Self-Regulation Skill Mastery: The VA DPP curriculum is iterative in nature as behavioral topics are introduced gradually over time and revisited in depth in later sessions. In contrast, VA MOVE! sessions focus on relatively independent topics that do not build on earlier topics. This focus on iterative skill building and mastery in the VA DPP may impact measures of self-regulation, self-monitoring, and willingness to self-monitor.

Intervention Intensity: The VA DPP includes 16 sessions over the first six months of the intervention while the VA MOVE! program generally includes eight to ten sessions over three months. The actual number of sessions offered differs across sites and is determined by the local VA MOVE! staff. Differences in total intervention exposure time may explain some of the differences in outcomes and sessions attended.

References

1. Deci EL, Ryan RM. Handbook of self-determination research. vol x, 470 p. Rochester, NY: University of Rochester Press; 2002.
2. Resnicow K, Davis RE, Zhang G, Konkel J, Strecher VJ, Shaikh AR et al. Tailoring a fruit and vegetable intervention on novel motivational constructs: results of a randomized study. Annals of behavioral medicine : a publication of the Society of Behavioral Medicine. 2008;35(2):159-69. doi:10.1007/s12160-008-9028-9.
3. Gabriele JM, Carpenter BD, Tate DF, Fisher EB. Directive and nondirective e-coach support for weight loss in overweight adults. Annals of behavioral medicine : a publication of the Society of Behavioral Medicine. 2011;41(2):252-63. doi:10.1007/s12160-010-9240-2.
4. Segar ML, Eccles JS, Richardson CR. Rebranding exercise: closing the gap between values and behavior. The international journal of behavioral nutrition and physical activity. 2011;8:94. doi:10.1186/1479-5868-8-94.
5. Segar ML, Eccles JS, Richardson CR. Type of physical activity goal influences participation in healthy midlife women. Women's health issues : official publication of the Jacobs Institute of Women's Health. 2008;18(4):281-91. doi:10.1016/j.whi.2008.02.003.
6. Segar ML, Updegraff JA, Zikmund-Fisher BJ, Richardson CR. Physical activity advertisements that feature daily well-being improve autonomy and body image in overweight women but not men. Journal of obesity. 2012;2012:354721. doi:10.1155/2012/354721.
