# Supplementary material for: Implementation and evaluation of the VA DPP clinical demonstration: protocol for a multi-site non-randomized hybrid effectiveness-implementation type III trial
Source: Implement Sci. 2015 May 12;10:68. doi: 10.1186/s13012-015-0250-0 (PMC4429938; doi:10.1186/s13012-015-0250-0)
Supplement: Additional file 2: — Research questions and RE-AIM measures. This file provides a detailed list of research questions and quantitative and qualitative measures for RE-AIM domains. [file 13012_2015_250_MOESM2_ESM.pdf]

VA DPP Study Protocol Additional File 2: Research Questions and RE-AIM Measures

Questions	Quantitative Measures	Qualitative Inquiry
**Reach**		
What proportion of patients is eligible?	Number eligible for the VA DPP/Number referred to VA MOVE!	
	Reasons for exclusion	
What proportion of patients enrolls (i.e., consents and attends at least one class)?	Number VA DPP enrollees/Number VA MOVE! enrollees/Number eligible enrollees	
What are characteristics of eligible patients that enroll versus eligible patients that do not enroll?	Proportion of enrollees of minority race and women enrolled in VA DPP/VA MOVE! compared to non-enrolled	Patient Level: What are reasons for enrolling or not enrolling?
What is reach into the target population?	Proportion of the target population referred to VA MOVE! X Proportion of eligible participants enrolled	
**Effectiveness**		
What is weight change at six months?	Change in weight at six months compared to baseline	Facility and Patient Level: What are staff and patients perceptions of why the intervention did or did not work (i.e., result in weight or A1c changes)?
What is A1c change at six months?	Change in A1c at six months compared to baseline	
What is level of engagement (attend at least four classes)?	Number VA DPP engaged/Number enrolled VA DPP	
	Number VA MOVE! engaged/Number enrolled VA MOVE!	
What are characteristics of those who engage versus those who do not?	Proportion of minority race and women engaged in VA DPP/VA MOVE! compared to non-engaged	Patient Level: What are reasons for engaging or not engaging?
What is level of participation (attend at least nine classes in six months)?	Number VA DPP participated/Number enrolled VA DPP	
	Number VA MOVE! participated/Number enrolled VA MOVE!	
What are characteristics of those who participate versus those who do not?	Proportion of minority race and women participated in VA DPP/VA MOVE! compared to non-participating	Patient Level: What are reasons for participating or not participating?
**Adoption**		
How many sites agree to implement the VA DPP?	Number of sites that agree to implement VA DPP	What are barriers and facilitators to sites agreeing to implement the VA DPP?
What proportion of sites complete VA DPP implementation?	Number sites that complete VA DPP implementation/Number agree to participate	

**Implementation**	
What are barriers and facilitators to implementing and administering VA DPP?	Note: Will use a focused set of CFIR constructs to systematically identify barriers and facilitators to implementation and administration and how they vary across sites:<br><br>Intervention Characteristics:<br>How complex is the program to implement and administer?<br>What is the perception of the evidence for VA DPP/VA MOVE?<br>What is the relative advantage of the VA DPP versus VA MOVE!?<br><br>Outer Setting:<br>Is the program perceived as meeting the needs of patients?<br>What is the influence of performance measures or other program initiatives coming from the national or regional level?<br><br>Inner Setting:<br>What are program team characteristics e.g., teamness, degree of interdisciplinary membership?<br>What is the quality and nature of communications and relationships across service lines (e.g., primary care and food & nutrition services)?<br>What is the level of cross-departmental coordination in implementation?<br>What is the nature and degree of leadership engagement?<br>How robust is the referral process?<br>What is the relative priority of implementation of VA DPP?<br>How compatible is VA DPP/VA MOVE! with values and other programs?<br>Do program goals align with organizational goals and is program data fed back for review?<br><br>Characteristics of Individuals:<br>What are attitudes and knowledge of key stakeholders?<br><br>Process:<br>How are providers and leaders engaged?<br>Does the VA DPP/VA MOVE! have a champion? What is their role and influence?<br>Are the programs being evaluated or tracked?<br>What is the influence of facilitating efforts of the research coordinating center?

To what extent is VA DPP/VA MOVE! delivered as intended?	Fidelity measures	
What is the level of consistency of VA DPP implementation across sites and time?	Fidelity measures by site/time<br><br>Number VA DPP components implemented/Total Number VA DPP components (weighted as appropriate)<br><br>Number VA DPP components implemented by time	
What is the cost of implementing and administering VA DPP?	CEA time logs, program costs, staff FTEs	
**Maintenance**		
What is weight change at 12 months?	Change in weight at six months compared to baseline	Facility and Patient Level: What are staff and patients perceptions of why the intervention did or did not work (i.e., result in weight or A1c changes)?
What is A1c change at 12 months?	Change in A1c at six months compared to baseline	
What is level of engagement (attend at least 4 classes)?	Number VA DPP engaged/Number enrolled VA DPP<br><br>Number VA MOVE! engaged/Number enrolled VA MOVE!	
What are characteristics of those who engage versus those who do not?	Proportion of minority race and women engaged in VA DPP/VA MOVE! compared to non-engaged	Patient Level: What are reasons for engaging or not engaging?
What is level of participation (attend at least nine classes in six months)?	Number VA DPP participated/Number enrolled VA DPP<br><br>Number VA MOVE! participated/Number enrolled VA MOVE!	
What are characteristics of those who participate versus those who do not?	Proportion of minority race and women participated in VA DPP/VA MOVE! compared to non-participating	Patient Level: What are reasons for participating or not participating?
What are prospects for continuing or restarting VA DPP?		Facility Level: What are staff perceptions of continuing or restarting the VA DPP?
