# Supplementary material for: Implementation and evaluation of the VA DPP clinical demonstration: protocol for a multi-site non-randomized hybrid effectiveness-implementation type III trial
Source: Implement Sci. 2015 May 12;10:68. doi: 10.1186/s13012-015-0250-0 (PMC4429938; doi:10.1186/s13012-015-0250-0)
Supplement: Additional file 3: — Staff interview guides. This file provides a copy of the master interview guides (early and late implementation) used to interview staff. [file 13012_2015_250_MOESM3_ESM.pdf]

**VA DPP Study Protocol Additional File 3: Staff Interview Guides**

**Pre-Implementation Interview Guide**

**The purpose of this interview is for us to learn about how implementation of the VA DPP has gone so far at your site. We would like to ask you about how your site got involved and the various activities that had to happen to get the VA DPP up and running.**

**Your perspective is important to us so that if the VA DPP is rolled out nationwide, we would be able to provide feedback as to what went well and what areas might need to be worked out at various sites.**

**Today, we will focus on pre-implementation of the VA DPP. Later in the year, we may ask you to do a second interview to gain insight on how the VA DPP is working at your facility.**

***First, I would like to ask you a few questions to help me understand your role at the VA.***

**Will you please describe your role within your facility?** **Background Information**

*PROBES*

- What is your title and role within your organization?
- What are your main responsibilities?
- To whom do you report (just position/title, not name)?

***Now I would like to ask you a few questions specific to the VA DPP.***

**What is your role in the VA DPP specifically?**

**How is your time allocated between your current role at the VA and the VA DPP? FTE?**

***I would like to hear about how you and others viewed the VA DPP before it was implemented at your site.***

**How did your site become involved with this intervention? How did you personally get involved?** **Intervention Source & Compatibility**

*PROBES*

- How was the decision made for your site to participate in the VA DPP?
- Who participated in the decision-making process?

**What kind of information or evidence were you aware of that showed whether the VA DPP would work?** **Evidence Strength & Quality**

*PROBES*

- o What is/was your perception of the evidence (e.g., does it make you think it will work for your patients? Or is it uncertain/unknown?)
- o Information from your own research, consensus guidelines, published literature, or other sources? From co-workers? From supervisors? From patients?

- To what degree did the evidence influence your opinion of the VA DPP before it was implemented?

**What kind of care management services are you already offering to patients who are overweight or obese at your site (e.g., by phone, internet, etc.)?** **Relative Advantage**

*PROBES*

- Please describe the different services/programs.
- To what extent are the existing services multi-disciplinary? To what extent do sessions build on one another?
- Are there other alternatives for encouraging patients to engage in physical activity at home?
- Do you have a prediabetes program currently at your site? If so, how is this program different from the VA DPP?

**Prior to the time your site decided to implement the VA DPP, did you see a need for a specific intervention program for Veterans with prediabetes? Why or why not?** **Patient Needs & Tension for Change**

*PROBES*

- Did other people at your site feel the VA DPP was needed?
- How well do you think the VA DPP will meet the needs of patients? (Improved outcomes?)
- Do you perceive the VA DPP as being a better alternative to other programs your VA offers to overweight or obese patients at risk for developing diabetes? Why or why not?

**Have you had any involvement with the VA MOVE! program at your VA?**

- If so, what was/is your role with VA MOVE!?
- If so, can you please compare the VA MOVE! program and the VA DPP?

***Now I would like to talk about the planning process and steps you took to implement the VA DPP at your site.***

**Can you describe the planning you did to get the VA DPP implemented?** **Planning**

*PROBES*

- Was/is there a clear aim/goals related to the implementation?
- Who was involved in the planning process?
- Were the appropriate people involved in the planning process? How engaged in the planning process were they?
    - Were there issues with staff buy-in?
    - Is there a champion (or opinion leader – someone strongly in favor who is influential) for the program? Do you think you need one?

- How did you track what tasks needed to be done? How did you decide who would do these tasks?
- How did you track the progress and status of tasks?
- What kind of communication/marketing activities were done?
- Is there an evaluation component built in?
  - How will you know if the program is successful or not?
- Did you attend the GLB/DPP training course in Pittsburgh?
  - If yes (and will teach the VA DPP), do you feel you are adequately prepared to teach the VA DPP after this course? Why/why not?
  - If yes (and you needed to gain understanding), do you feel like you understand the VA DPP after taking this course? Why/why not?

**What issues did you encounter when planning the implementation of the VA DPP?**

*PROBES*

- Hiring VA DPP employees
  - Are you using existing staff at your site? If so, how is this working with their current job requirements?
  - If you had to go outside the VA, how has the hiring process gone?
- Recruiting/referrals
- Space
- Scheduling
- Anything else?

**What is the process for identifying Veterans with prediabetes?**

*PROBES*

- How did you identify Veterans' eligibility criteria?
  - A1c, fasting glucose
  - Distance factor
    - Do you think Veterans would attend the VA DPP if they live > 60 mins/miles? Why/why not?
  - Metformin
  - Pregnancy
  - Eating disorders
- Describe if any processes were changed or added to determine eligibility such as working with the lab, physician referrals, CPRS templates, etc.
- What issues did you encounter?

**What connections outside of your own department did you have to make (CPRS people, HR, lab personnel, physicians, etc) in order to implement the VA DPP? What are/were challenges?**

**As you move toward implementation of the VA DPP at your site, have you had to change any aspects of the VA DPP in order to get it implemented at your site? If so, what were they?**

*PROBES*

- Is there anything that can be done to make the DPP/GLB program fit better (more flexibility) at your site?

**What kinds of support are/were you hoping to get from the research coordinating center? Were these needs met? As we move forward, how can the research coordinating center support the implementation of the VA DPP at your site?**

*PROBES*

- Do you regularly sit in on the bi-weekly telephone conference calls for the VA DPP all-team meeting?
- Do you find the bi-weekly telephone conference calls to be useful? Why or why not?
- Is the timing, format, content, mode (e.g., phone, LiveMeeting) of these meetings good? Any suggestions to improve these?

***Now I would like to discuss how your site plans to implement the VA DPP class.***

**Do you anticipate difficulty filling a VA DPP class with 20 patients every 2 months? Why/why not?**

**How are you handling patients that are VA DPP eligible but the VA DPP class is full?**

*PROBES*

- How will you handle patients that want to wait for a new VA DPP class (but will be referred to VA MOVE!)?

**Are the VA DPP/GLB Program materials helpful to you?** **Design Quality & Packaging**

*PROBES*

- What is your experience with the supporting materials for the VA DPP? E.g., the VA DPP/GLB Leader Guide; Patient Workbooks; DVDs; Nutrition Tracker; Calorie King; Exercises.
- Are there materials you feel are important that are not part of the VA DPP? If so, what are they?
    - Are there materials that the VA DPP/GLB program offers but are not provided by the research coordinating center that you think are important?
        - Exercise DVD, some of the Misc Handouts (Handouts for Southerners, Certificate of Completion)
        - Did you make copies of anything at your site?
- If you attended the GLB training course in Pittsburgh, did you ever utilize the support staff there?

  - What was your experience?
  - For example: timeliness getting back with you, answered your questions satisfactorily, etc.

**Will you please describe the physical space configuration used for the VA DPP?** **Available Resources**

*PROBES*

- What kind of space will be/is used for VA DPP classes?
- Where will/are the classes located? Onsite? Offsite?
- Please describe any issues you had finding space to hold the VA DPP class.

**What level of involvement do leaders at your facility have with the VA DPP?** **Leadership Engagement**
*(**Who is the interviewee referring? ACOS, Director of Primary Care, COS, Chief of Food & Nutrition, etc)*

*PROBES*

- Do they know about the VA DPP? (who is involved; e.g., Primary Care Director, ACOS, your service chief?)
- What kind of support do they give you? Specific examples. (.e.g moral support; help with solving problems, asking you about how things are going?) Do your interactions tend to be formal (via email through the "chain of command" or informal (e.g., ad hoc hallway conversations)?
- Do they provide any feedback? What kind of feedback?
- Do you feel they are too involved or not involved enough? Why?
- Has the program been on the agenda at any meetings? (This could be under the communications/marketing section but it could also be here since it's usually some clinical leader who controls the agendas)

**Do you feel you have sufficient information and resources to make the VA DPP work?** **Access to Knowledge and Information & Available Resources**

*PROBES*

- From your facility?
- From NCP staff?
- From the research coordinating center?

**And now, summing up your implementation of the VA DPP experience thus far:**

On a scale of 0-10, how difficult has it been so far to implement the VA DPP?
0 is easy (no difficulties) → 10 (extremely difficult; it couldn't get any more difficult)

Why?

On a scale of 0-10, how difficult do you think it will be to implement the VA DPP as you move ahead?
0 is easy (no difficulties) → 10 (extremely difficult; it couldn't get any more difficult)

Why?

How successful has the program been so far? 0 failure → 10 it couldn't be any more successful

**Who else do you think we should interview at this point? (IT, etc)**
Note: Only ask if you don't think we have everyone covered

## Post-Implementation Interview Guide

**Introductions**

My name is ________ and I am a researcher working on the project entitled, “VA Diabetes Prevention Program Demonstration Project.”

Before we begin, I would like to take a minute to explain why I am inviting you to participate and what I will be doing with the information you provide to me. Please stop me at any time if you have any questions. After I’ve told you a bit more about my project, you can decide whether or not you would like to participate.

The purpose of this interview is to help us understand your experience implementing and administering the VA DPP at your facility. I am interested in hearing about contextual factors that facilitated or hindered implementation efforts. Your input will help us to evaluate the program and improve it for the future.

Participation is purely voluntary. If you agree to participate in this interview, you will be asked questions related to your experiences in the VA DPP. The interview should take approximately 30-60 minutes and will be audio taped so that we do not miss anything that you have to say.

If at any time and for any reason, you would prefer not to answer any questions, please feel free not to answer. If at any time you would like to stop participating, please tell me. We can take a break, stop and continue at a later date, or stop altogether. You will not be penalized in any way for deciding to stop participation at any time.

I would like to record this interview to make sure that I remember accurately all the information you provide. However, if you do not want to be recorded, I will only take notes during the interview.

Any information you provide will be handled in a confidential manner. Only people working on this study will use the interview recordings. We will take steps to ensure your answers stay confidential.

Your name will not appear on any of the transcripts. Each audio file will be labeled only with a study ID number, which will be linked to the name of the facility in a “crosswalk” file. The crosswalk file will be kept on a secure drive separate from the transcripts. The audio file will be maintained on our secure server. No site or individual names will appear on any of the transcripts. The interview transcript will be given a study ID number, again linked only to the name of the facility, and any personal references that would identify any individuals will be removed.

If you have questions, you are free to ask them now. If you have questions later, you may contact me at 1-800-753-3357.

Are you interested in participating in this study?

Great, I'm going to turn on the DVR and record you giving consent and then we will get started.

If the participant agrees to participate, start the recording and begin the interview. *Interviewers please make a note to probe for the title of any person mentioned by the interviewee but discourage the use of individual names. In addition, please state your name, the participant's ID number, and the date of the interview at the beginning of the recording and the participant giving consent to be interviewed.*

| CFIR Construct | Closed-ended Questions Scale: 1-strongly disagree, 2-disagree, 3-neutral, 4-agree, 5-strongly agree, or unknown/not applicable. | Open-ended questions |
|---|---|---|
| **Introduction:** Thank you for taking time to be interviewed today. We may have asked you some of these questions the last time we spoke with you, but we want to ask them again to be sure that we captured the correct information. | | |
| Role in Facility, Role in the VA DPP | None | For the record, can you confirm your roles and responsibilities in [site]?<br>What is your role in the VA DPP?<br>• How much of your time has been dedicated?<br>Has that changed since the beginning of the VA DPP? |
| General Assessment, Milestones | None | Can you describe your overall experience related to the VA DPP?<br>• What major milestones have you achieved?<br>• Can you describe the implementation process?<br>• Can you describe how you have run the program?<br>• In general, how is the VA DPP going? |
| Program Administration | None | Can you walk us through how the VA DPP runs on a day-to-day basis?<br>• How are patients screened for the VA DPP eligibility?<br>• How are patients enrolled?<br>o [If through VA MOVE!] Who are major sources of referrals to VA MOVE!?<br>o Does your site use the obesity screening clinical reminder?<br>o How does this influence the number of referrals?<br>• Has this changed from the beginning? Why?<br>• What are your recommendations for identifying and referring patients to the VA DPP?<br>o Should the VA DPP continue to receive referrals through VA MOVE! or should the VA DPP have a different referral process? |

| CFIR Construct | Closed-ended Questions Scale: 1-strongly disagree, 2-disagree, 3-neutral, 4-agree, 5-strongly agree, or unknown/not applicable. | Open-ended questions |
|---|---|---|
| | | ○ Would it be beneficial to have a clinical reminder for BMI & A1C and patients with both would be referred to the VA DPP?<br>• How do patients get started in the VA DPP?<br>○ Is there an orientation session? How often are they held? What is covered?<br>• Does anyone make telephone calls to participants? [If yes] For what reason?<br>• When and how are participants documented in CPRS?<br>○ Health factor assignment, classes, no shows, calls?<br>• Have there been any issues getting materials for the VA DPP participants (class handouts, pedometers, kitchen scales, water bottles)? |
| | Now, I'm going to ask you a series of close-ended questions. There is no right or wrong answer to any of these questions. The possible responses for each item are: 1-strongly disagree, 2-disagree, 3-neutral, 4-agree, 5-strongly agree, or unknown/not applicable. Give the response that fits most closely with your perception. We will ask open-ended question about your responses so we can better understand how the VA | **All closed-ended questions may be followed with a one or more of the following probes based on the question and the response:**<br>• **Can you explain your response?**<br>• **What were the key factors that contributed to that (high/low) response?**<br>• **What could have been done to help make that better?**<br>• **What would need to change to make that better?** |

| CFIR Construct | Closed-ended Questions Scale: 1-strongly disagree, 2-disagree, 3-neutral, 4-agree, 5-strongly agree, or unknown/not applicable. | Open-ended questions |
|---|---|---|
| | DPP was experienced at your site. | |
| **DPP Implementation and Intervention Characteristics**: The first questions I will ask are about the VA DPP within your facility. | | |
| Planning | We had a clearly communicated implementation plan for the VA DPP. | Can you describe the plan you had and how it helped or hindered implementation?<br>• Was it a formal plan?<br>• Was it written down somewhere?<br>• Was it updated?<br>• Did you feel it was realistic? Overly simple? Overly complicated?<br>• Was it missing any major component?<br>• Do you have any advice for future sites when planning the VA DPP implementation? |
| Complexity | The VA DPP implementation was very complex and difficult. | What was the most difficult or complicated aspect of implementation?<br>What was the easiest aspect of implementing the VA DPP? |
| Evidence Strength and Quality | Overall, key clinical staff at your facility feels that the evidence base demonstrates that DPP is effective. | What evidence did you have about DPP effectiveness? |
| Adaptability | The VA DPP is highly adaptable and is thus suited for a wide variety of settings in the VA. | What aspects of the VA DPP are the most important to be able to adapt?<br>If you were able to change one thing related to DPP, what would it be?<br>• How important is this? |
| | There are components of DPP that are too rigid and contributed to problems or made | What aspects of DPP were too rigid?<br>• How would you alter those? |

| CFIR Construct | Closed-ended Questions Scale: 1-strongly disagree, 2-disagree, 3-neutral, 4-agree, 5-strongly agree, or unknown/not applicable. | Open-ended questions |
|---|---|---|
| | implementation more difficult. | |
| Compatibility | The VA DPP aligns with what clinicians believe about how their patients can best be served.<br><br>The VA DPP is compatible with other similar clinical treatments or programs.<br><br>The VA DPP is well integrated into the operations of the facility. | |
| Relative Advantage | The VA DPP appears to have many more advantages than disadvantages. | Can you describe the advantages and disadvantages of the VA DPP compared to other programs? |
| | The VA DPP appears to have advantages compared to other similar programs like VA MOVE!. | How does the VA DPP differ from other similar programs like VA MOVE!?<br>Should the VA DPP be part of VA MOVE! or a different program?<br>• Should the VA DPP curriculum be part of VA MOVE! curriculum? |
| Relative Priority | The VA DPP takes a backseat to other initiatives or programs in your facility. | What do you perceive to be the highest priorities of leadership? |

| CFIR Construct | Closed-ended Questions Scale: 1-strongly disagree, 2-disagree, 3-neutral, 4-agree, 5-strongly agree, or unknown/not applicable. | Open-ended questions |
|---|---|---|
| **Implementation Leaders and Facility Climate:** Now I want to ask you some questions about the people implementing the VA DPP and how they work together. | | |
| FAIIL | There has been a clearly designated person or team leading implementation and administration of the VA DPP. | Who are these people? [Roles only] |
| | The VA DPP implementation leader(s) have the necessary qualities and skills to implement the program. | Based on your experience, what qualities or skills are most important for this/these leader(s) to have? |
| | The person or team leading the VA DPP has had sufficient authority to do what is/was necessary in order for the VA DPP to be successful. | |
| Networks and Communication | The people involved with the VA DPP have strong and productive working relationships. | Can you describe these working relationships generally?<br>• What is most challenging?<br>• What works well?<br>How does the team communicate?<br>• Meetings? Email? Frequency of meetings? |

| CFIR Construct | Closed-ended Questions Scale: 1-strongly disagree, 2-disagree, 3-neutral, 4-agree, 5-strongly agree, or unknown/not applicable. | Open-ended questions |
|---|---|---|
| | It was/is a challenge to work with people in other departments to implement and administer the VA DPP. | What units/services did/do you work with to implement the VA DPP?<br>• Can you describe how that worked?<br>• How would you characterize your communication with these units/services? |
| Learning Climate | People felt free to speak up if they saw something wrong while implementing the VA DPP.<br><br>People viewed the VA DPP as a new initiative that would go away after a while.<br><br>Mistakes or problems related to DPP led to positive changes or adjustments. | Can you think of an example when you or another team member planned something that did not work?<br>• If yes, what was the reaction of the team? Leadership?<br>• Did they help you come up with any solutions?<br>• If no, why not?<br>Who did you talk to most at your site about issues you had with implementation?<br>• Did they offer any suggestions?<br>If other staff members offered suggestions you knew would not work, were you able to speak up? |
| Culture | This question is a bit different in terms of the responses. How would you best characterize the overall culture at your site? I'll describe four different types of | How does/did aspects of this culture generally help or hinder the implementation and administration of the VA DPP? |

| CFIR Construct | Closed-ended Questions Scale: 1-strongly disagree, 2-disagree, 3-neutral, 4-agree, 5-strongly agree, or unknown/not applicable. | Open-ended questions |
|---|---|---|
| | culture, please select the one that seems to fit most closely with yours.<br><br>**Team (Clan) Culture (Flexible, Internal Focus):** A friendly workplace where leaders act like mentors, facilitators, and team-builders. There is value placed on long-term development and doing things together.<br>**Hierarchical (Hierarchy) Culture (Control, Internal Focus):** A structured and formalized workplace where leaders act like coordinators, monitors, and organizers. There is value placed on incremental change and doing things right.<br>**Entrepreneurial (Adhocracy) Culture (Flexible, External** | |

| CFIR Construct | Closed-ended Questions Scale: 1-strongly disagree, 2-disagree, 3-neutral, 4-agree, 5-strongly agree, or unknown/not applicable. | Open-ended questions |
|---|---|---|
| | **Focus):** A dynamic workplace with leaders that stimulate innovation. There is value placed on breakthroughs and doing things first.<br>**Rational (Market) Culture (Control, External Focus)**: A competitive workplace with leaders like hard drivers, producers, or competitors. There is value placed on short-term performance and doing things fast. | |
| **Resources:** Now I want to ask you some questions about the resources that you needed to implement and administer the VA DPP. | | |
| External Change Agents | The research coordinating center provided excellent facilitation and support to implement and administer the VA DPP.<br><br>We could have | What were the most important aspects of this support?<br>• Implementation planning<br>• Problem solving<br>• Availability/accessibility<br>• Respect/trust<br>• Reliable knowledge<br>What could be improved?<br>How essential is this support for future sites? |

| CFIR Construct | Closed-ended Questions Scale: 1-strongly disagree, 2-disagree, 3-neutral, 4-agree, 5-strongly agree, or unknown/not applicable. | Open-ended questions |
|---|---|---|
| | implemented the VA DPP without the help and support from the research coordinating center. | What are the most essential/minimum components necessary for successful implementation? |
| Available Resources | The program has adequate staff to implement and administer the VA DPP.<br><br>The program has adequate space to implement and administer the VA DPP. | *Do not include consideration of materials from NCP.*<br>What were the available resources that contributed most significantly to being able to implement the VA DPP? Administer the VA DPP?<br>What were the biggest resource constraints you encountered while implementing and administering the VA DPP?<br>What kind of space was used for patient orientation and program enrollment? Screening? The VA DPP sessions?<br>• What was the location relative to other related programs, e.g., VA MOVE!? |
| | You have had insufficient time to dedicate to the VA DPP. | How did/do your other VA work responsibilities fit in with the VA DPP?<br>• Are there other clinical priorities that compete for your time over the VA DPP? Relative Priority<br>• [If yes] Can you tell me more about these priorities?<br>• Did you receive relief from other duties?<br>• Do you receive help from staff from other departments? |
| Access to Knowledge and Information | We had sufficient support in terms of training and education to implement and administer the VA DPP. | Did you have sufficient access to information about the VA DPP from the research coordinating center? From the Diabetes Prevention Support Center? |
| Available Resources | We had sufficient materials and resources | *Materials include: VA DPP/GLB Leader Guide, Patient Handouts, Pedometers, DVDs, Nutrition Tracker, Calorie King, Exercises.* |

| CFIR Construct | Closed-ended Questions Scale: 1-strongly disagree, 2-disagree, 3-neutral, 4-agree, 5-strongly agree, or unknown/not applicable. | Open-ended questions |
|---|---|---|
| | to implement and administer the VA DPP. | What materials and resources were most helpful?<br>• How were they used?<br>• Are there materials that you didn't have that you feel would have improved the VA DPP? (Different pedometer, exercise DVD, resistance bands.) |
| Design Quality and Packaging | The packaging and content of the VA DPP materials and components was excellent. | Could these materials be improved? How? |
| **Engagement:** Now I want to ask you some questions about how implementation leaders engaged facility staff in the VA DPP. | | |
| Leadership Engagement | Leaders have openly endorsed and supported the VA DPP in visible ways.<br><br>*Leadership includes the Chief of Staff, Primary Care Director/ACOS, Facility Director and other supervisors, not implementation leaders.* | How familiar are leaders with the VA DPP?<br>How have leaders demonstrated their (lack of) support for the VA DPP?<br>• Do you have an example of when you approached a leader to get help solving a problem? What happened?<br>What leadership actions were necessary to implement the VA DPP (i.e. if they hadn't happened, the program would have failed)?<br>• Do you have specific examples, e.g., dedicated time, staff hiring approvals, getting the VA DPP on key agendas, help solving a specific problem, moral support?<br>What were strategies you used to get leaders on board and supportive of the VA DPP? |
| | Leaders established clear goals for the VA DPP. | What kinds of goals have been communicated by your facility's leaders related to the VA DPP? |
| | Leaders provided staff with needed processes | |

| CFIR Construct | Closed-ended Questions Scale: 1-strongly disagree, 2-disagree, 3-neutral, 4-agree, 5-strongly agree, or unknown/not applicable. | Open-ended questions |
|---|---|---|
| | and tools to monitor the progress of the VA DPP. | |
| | Leaders have indicated that the VA DPP failure is unacceptable. | What kind of feedback or questions do you hear from leaders about the VA DPP's success or performance? |
| Engaging: Opinion Leaders | Influential clinicians have openly endorsed and supported the VA DPP.<br><br>*Influential clinicians are people who have informal (positive or negative) influence on the attitudes and beliefs of their colleagues.* | Who are the most important people to engage to help ensure the VA DPP's success in your site?<br>• Formal clinical or administrative leaders?<br>• VA MOVE!?<br>• PCPs?<br>• Care Managers |
| Engaging: Staff | Information about the VA DPP is communicated effectively to key staff outside of the VA DPP/VA MOVE! at my site. | What is your communication strategy?<br>• How do you communicate with staff? Personal contact? Formal meetings?<br>• Do you have ongoing communication?<br>• Do you use different strategies with different staff? Why?<br>• Are there flyers around your site about the VA DPP? |
| Champion | There has been a champion for the VA DPP who works well with key staff. | What is his/her role?<br>Can you describe their role related to the VA DPP?<br>• What makes them a champion? |

| CFIR Construct | Closed-ended Questions Scale: 1-strongly disagree, 2-disagree, 3-neutral, 4-agree, 5-strongly agree, or unknown/not applicable. | Open-ended questions |
|---|---|---|
| **DPP Patients:** Now I want to ask you some questions about patient experiences with the VA DPP. | | |
| Engaging: Patients | Information about the VA DPP is communicated effectively to patients who may be candidates for the VA DPP. | To what extent are patients aware of the VA DPP?<br>What is your communication strategy?<br>• What worked best?<br>• How do patients respond to information about the VA DPP? VA MOVE!? |
| Patient Needs and Resources | The VA DPP meets the needs and preferences of your patients. | What kind of impact did the VA DPP have on patients?<br>What feedback have you heard from patients?<br>• Anecdotal stories of success or failure?<br>What is unique about patients at your site?<br>• Were there particular types of patients (e.g., older, women) who preferred the VA DPP or VA MOVE!?<br>Why do you think patients chose to participate in the VA DPP?<br>• Motivated to improve their diet or eating habits?<br>• Motivated to be physically active?<br>• Motivated to prevent diabetes or improve their health?<br>What are the barriers for patients enrolling or participating in the VA DPP?<br>What are your perceptions of the reasons patients did not show and/or canceled their VA DPP appointment(s)? |
| **Reflecting and Evaluating and Recommendations**: We are nearing the end of the interview, now I want to ask you about how you evaluate the success of the VA DPP and if you think it should be implemented in other facilities. | | |
| Reflecting and Evaluating | Status of the VA DPP has been monitored consistently over the course of the evaluation period. | Can you describe how you track the progress of the VA DPP?<br>• What measures and sources do you use?<br>○ Recruiting targets? Enrollments? Participation rates? Anecdotal data?<br>○ VA DPP v. VA MOVE!<br>○ Elicit input from patients? Staff?<br>To what extent is progress communicated to staff? How? Who? |

| CFIR Construct | Closed-ended Questions Scale: 1-strongly disagree, 2-disagree, 3-neutral, 4-agree, 5-strongly agree, or unknown/not applicable. | Open-ended questions |
|---|---|---|
| | Status of the VA DPP is regularly shared with other staff | Who is responsible for tracking and reporting the VA DPP progress?<br>When and how do you take time to report and/or review the VA DPP progress?<br>Is this data used to make program decisions or make a business case to e.g., get more resources? |
| Knowledge and Beliefs about the Intervention | The VA DPP is a successful program. | What specifically made the VA DPP successful or unsuccessful?<br>What do you measure to determine whether the VA DPP is successful?<br>• Weight loss? Satisfaction? Hospital utilization? Other outcomes? |
| | The VA DPP should be continued at your site. | What are the prospects for sustaining the VA DPP at your site?<br>What type of justification would you need to show in order to keep this intervention going over the long-term? |
| | The VA DPP should be rolled out to other VA sites. | Do you have any specific suggestions that we haven't already covered for other sites that have not yet started implementing the VA DPP? |
| Available Resources | You have everything you need to keep the VA DPP up and running for the foreseeable future. | What resources would be needed to keep the VA DPP going on a long-term basis? |
| Organizational Incentives & Rewards | Leaders hold/held staff accountable for the success of the VA DPP. | What incentives did people have to implement the VA DPP?<br>• What incentives do you have to keep the VA DPP going?<br>• Is it considered in evaluations?<br>What other kinds of incentive programs would be needed for other sites to implement the VA DPP? External Policy & Incentives<br>• VA performance measures?<br>• The CDC's recognition program? |
| **CLOSING:** Those are all the questions I have for you. Is there anything I've missed or anything you'd like to add? If no, thank you for taking time to speak with me today! | | |
