# Supplementary material for: Implementation and evaluation of the VA DPP clinical demonstration: protocol for a multi-site non-randomized hybrid effectiveness-implementation type III trial
Source: Implement Sci. 2015 May 12;10:68. doi: 10.1186/s13012-015-0250-0 (PMC4429938; doi:10.1186/s13012-015-0250-0)
Supplement: Additional file 4: — Session delivery fidelity checklists for the VA DPP and VA MOVE!. This file includes copies of fidelity checklists used to assess VA DPP fidelity to the GLB program and to compare VA DPP sessions to VA MOVE! sessions. [file 13012_2015_250_MOESM4_ESM.pdf]

VA DPP Study Protocol Additional File 4: Session Delivery Fidelity Checklists for VA DPP and VA MOVE!

VA DPP Fidelity Checklist
Session Five: Tipping the Calorie Balance

Site:____________________	Cohort:________________________	Group Leader:_________________
Evaluator: _______________	Attendance:____________________	Date:__________________________
Scheduled Session Begin Time: __________________	Scheduled Session End Time: __________________	
Actual Time Session Began:_____________________	Actual Time Session Ended:____________________	

Instructions:

1. Review the session in the GLB workbook and the fidelity checklist prior to the session.

2. Complete *Part One: Food Dairies* before Session 5 begins based on the coach's comments in the food diaries collected in Session 4. *We want to capture the overall tone of the of the coach's comments; therefore a smiley face ☺ can be counted as a positive comment.*

3. Complete *Part Two: Session Content* during the session. Session activities were fully completed, partially completed, or not completed. **Use the comment section to explain the rating as needed, especially when an activity was partially completed (i.e. what aspect was complete, what aspect was incomplete). Please fill in each box in the table, including boxes next to sub questions.**

   Fully Completed Activities: this activity was fully completed with all participants

   Partially Completed Activities: this activity occurred, but did not occur with all group members or all of the time (i.e. group leader facilitated the discussion but only among certain members of the group or s/he began to demonstrate with an individual participant but did not follow through to adequate resolution)

   Not Completed Activities: this activity did not occur with any participants

4. Complete *Part Three: Session Principles* after the session based on the content checklist and your personal experience in the session. You will be assessing:

   Delivery of Educational Content
   Review of Goal Progress
   Goal Setting
   Group Cohesion
   Interventionist Characteristics

Part One: Food Dairies
Number of participants that attended Session 4.
____

Number of food diaries turned in at Session 4.
____

The group leader writes in green pen in:

- ☐ None of the diaries
- ☐ A few of the diaries (about ¼ of the diaries)
- ☐ About ½ of the diaries
- ☐ Most of the diaries (¾ of the diaries)
- ☐ All of the diaries

The group leader uses the *sandwich method* (one positive comment, one suggestion, and one positive comment) to comment on the front of:

- ☐ None of the diaries
- ☐ A few of the diaries (about ¼ of the diaries)
- ☐ About ½ of the diaries
- ☐ Most of the diaries (¾ of the diaries)
- ☐ All of the diaries

Inside each food diary, the group leader **generally** writes ____ positive comments or notations, i.e., a smiley face.
[Please note in the margin if there is an outlier to the general trend, i.e. most diaries have 4-6 comments but one diary didn't have any comments.]

- ☐ 0
- ☐ 1-3
- ☐ 4-6
- ☐ 7 or more

Inside each food diary, the group leader **generally** writes ____ suggestions for improvement.
[Please note in the margin if there is an outlier to the general trend, i.e. most diaries have 4-6 suggestions but one diary didn't have any suggestions.]

- ☐ 0
- ☐ 1-3
- ☐ 4-6
- ☐ 7 or more

Part Two: Session Content

**Principle**	**Session Content**	**Checklist**<br>0 = not completed<br>1 = partially completed<br>2 = fully completed	**Comments**
**Process**	Group leader elicits a report of any study or non-study related medical events from the group.		
**Goal Setting: Reviewing Progress**	Group leader weighs each patient privately and updates logs and graphs.<br>▪ If the patient has lost weight: Group leader congratulates participant, but stresses the fact that he or she must already be making some changes in behavior.<br>▪ If the participant has not lost weight: Group leader mentions it but stresses that little by little the numbers will change as the participant makes behavior changes.		
	Group leader asks if the participants weighed themselves at home; mentions that home weights and weights taken at the session may differ because scales differ, but the trend in weight change over time should be the same.		
**Process**	Group leader collects Keeping Track booklets and distributes blank booklets and booklets with comments to participants.		
**Process**	Group leader distributes session handouts.		

Principle	Session Content	Checklist<br>0 = not completed<br>1 = partially completed<br>2 = fully completed	Comments
**Goal Setting: Reviewing Progress**	Group leader reviews the last session, including home activities, and the group discusses successes and difficulties in meeting the program goals.<br>▪ Were you able to follow your activity plan?<br>▪ Were you able to make the active lifestyle choices you had planned?		
**Skill Development: Problem Solving**	Group leader praises all progress and problem solves with participants.		
**Goal Setting: Reviewing Progress**	Graphing Activity<br>Group leader distributes How Am I Doing? graph for activity and demonstrates how to use the graph. Group leader has participants graph their activity from the previous week and answers any questions about how to use the graph. Group leader tells the participants that they will use the graph to see at a glance the progress they have made over time and how they are doing compared to their activity goals.		
**Skill Development: Education**	Group leader explains how healthy eating and being active are related in terms of calorie balance: Group leader reviews that VA DPP involves two types of lifestyle changes, healthy eating and being active, and explains that these changes help prevent diabetes and lower the risk of developing other diseases. However,		

**Principle**	**Session Content**	**Checklist**<br>0 = not completed<br>1 = partially completed<br>2 = fully completed	**Comments**
	they are also important because they are related to weight loss and what's called the "calorie balance."<br>Group leader reviews:<br>▪ Calories in food come from fat, carbohydrates, protein, or alcohol. Other ingredients in food, like vitamins, minerals, and fiber don't have calories.<br>▪ The number of calories in any food depends on what's in the food. Fat is the most concentrated in calories, with 9 calories per gram. That's more than twice the number of calories in carbohydrates and protein and two more grams than alcohol.<br>▪ Calories measure the energy you use or burn.<br>▪ You use calories for just staying alive (breathing) and any activity you do.<br>▪ The number of calories you use in a certain activity depends on several things, including the type of activity, the amount of time you are active, and how much you weigh.<br>▪ In general, 1 mile of brisk walking (15-20 minutes) burns about 100 calories.		
	Group leader explains how calorie balance is related to weight loss: weight is determined by the balance between food (calories in) and activity (calories out).		

**Principle**	**Session Content**	**Checklist**<br>0 = not completed<br>1 = partially completed<br>2 = fully completed	**Comments**
	Eating and activity work together to determine how much you weigh. To lose weight, it's best to eat less and be more active.<br>▪ Weight Maintenance: calories in from food equal calories out from activity.<br>▪ Weight Gain: calories in from food are higher than calories out from activity. Either calories have increased or activity has decreased or both.<br>▪ Weight Loss: calories in from food are lower than calories from activity. You've eaten less food or calories or you've done more activity or both.<br>▪ Weight Maintenance at a Healthier Weight: you have developed new eating habits and new activity habits and they are balanced again. This is what happens when you lose weight and keep if off.		
	Group leader explains calorie requirements for weight loss: 1 lb of body fat stores about 3,500 calories and slow, steady weight loss (1-2 lbs per week) is the best way to lose weight.<br>▪ In order to lose 1 pound in a week, you need to tip your energy balance by 3,500 calories in a week, or 500 calories per day for 7 days.<br>▪ In order to lose 1.5 pounds in a week, you need to		

**Principle**	**Session Content**	**Checklist**<br>0 = not completed<br>1 = partially completed<br>2 = fully completed	**Comments**
	tip your energy balance by 5,250 calories in a week, or 750 calories per day for 7 days.<br>▪ In order to lose 2 pounds in a week, you need to tip your energy balance by 7,000 calories in a week, or 1,000 calories per day for 7 days.		
**Goal Setting: Reviewing Progress**	Group leader asks: What changes have you made to be more active? Allows participants to record some of the changes they have made and asks volunteers to share. Group leader praises and encourages the maintenance of those changes.		
	Group leader asks: What changes have you made to eat less fat and fewer calories? Allows participants to record some of the changes they have made and asks volunteers to share. Group leader praises and encourages the maintenance of those changes.		
	Group leader asks: Have you lost weight? Have these changes tipped the calorie balance?<br>▪ Participants refer to How Am I Doing? graph and record starting weight, current weight, and expected weight on worksheet.<br>▪ Group leader asks participants to check their weight trajectory. If the participant:		

Principle	Session Content	Checklist<br>0 = not completed<br>1 = partially completed<br>2 = fully completed	Comments
	• Stayed at the same weight or gained some weight: Group leader explains that to lose weight, they need to try something else to tip the calorie balance. Group leader tells the participant that they will work together to find out what will work better for the participant.<br>• Lost some weight, but not as much as expected: Group leader praises progress and explains that to lose more weight, they need to try something else to tip the calorie balance further.<br>• Lost as much weight as expected or more: Group leader praises progress and explains that if they keep tipping the balance, they will keep losing weight.		
**Skill Development: Self-Monitoring**	Home Activity: Group leader asks participants to continue to track their weight, eating, and activity.		
**Goal Setting: Goal Commitment**	Home Activity: Group leader assigns 90 minute activity goal; participants are encouraged to set aside one block of time each day or use 10-15 minutes that open up during the day to be physically active.		

Principle	Session Content	Checklist<br>0 = not completed<br>1 = partially completed<br>2 = fully completed	Comments
**Skill Development: Education**	If weight loss has not been as expected:<br>▪ Lowering fat and calorie goals: Group leader explains that participants may be eating more fat grams and calories than they realize, or that their goals may be too high for them. If they are not losing 1-2 lbs per week, group leader explains that they can lower their calorie goals by 300 calories per day. Group leader stresses that it is important they do not go below 1200 calories per day and reminds them to watch out for foods that are high in calories and fat, to record everything, and to watch portion sizes.<br>▪ Structured meal plan: Group leader explains that participants won't be faced with a lot of decisions and temptations about food. It will also make it easier to record what they eat. Group leader offers them two GLB plans as well as a shopping list and two recipes.		
**Goal Setting: Goal Commitment**	Group leader has participants record lower goals or follow a structured meal plan with a specific number of calories.<br>Group leader asks participants: What do you think will be most helpful? Lowering fat grams and calories or following a structured meal plan? Or would you like to		

**Principle**	**Session Content**	**Checklist**<br>0 = not completed<br>1 = partially completed<br>2 = fully completed	**Comments**
	try both?		
**Process**	Group leader distributes meal plans to participants who request one and provides tailored versions as appropriate. Group leader answers any questions or concerns the participants have regarding following the meal plans. Group leader presents the meal plans as a flexible model which the participants can use as a guideline.		
**Skill Development: Education**	Group leader double checks and corrects portion estimation using food models or real food.		
**Goal Setting: Goal Commitment**	Group leader has participants complete the “To Do” worksheet if they have not already done so during the session.		
**Process**	Group leader ends session and reminds participants that they will keep working together to try to tip the calorie balance and see how the scales respond.		
**What activities or additional topics were added to any aspect of the session?**			

Principle	Session Content	Checklist<br>0 = not completed<br>1 = partially completed<br>2 = fully completed	Comments
**Additional comments about the session?**			

Part Three: Session Principles

Delivery of Educational Content
Group leader elicited discussion of the educational content in order to help participants develop a self-management skill or change cognitions.

Group leader delivered approximately ______% percent of the educational content to the participants.

Review of Goal Progress
Group leader prompted review of goal progress and attainment.

1<br>Strongly Disagree	2	3	4	5	6	7<br>Strongly Agree

Group leader elicited discussion of successes and challenges since the last session.

1<br>Strongly Disagree	2	3	4	5	6	7<br>Strongly Agree

Group leader initiated problem solving when necessary to address challenges since the last session.

1<br>Strongly Disagree	2	3	4	5	6	7<br>Strongly Agree

Goal Setting
Diabetes prevention was discussed as a goal of the group.

1<br>Strongly Disagree	2	3	4	5	6	7<br>Strongly Agree

Group leader presented standardized goals (i.e. everyone had the same goal to complete the following week) to the participants and asked them to commit to a goal.

1<br>Strongly Disagree	2	3	4	5	6	7<br>Strongly Agree

Group Cohesion
Group identity includes having a diagnosis of prediabetes.
[*Coach and/or group members acknowledge a commonality among members in being diagnosed with prediabetes.]*

1<br>Strongly Disagree	2	3	4	5	6	7<br>Strongly Agree

There were new members present in the session.
☐ No
☐ Yes

VA DPP Study Protocol Additional File 4: Session Delivery Fidelity Checklists for VA DPP and VA MOVE!

Group members communicated easily with one another.

1 Strongly Disagree	2	3	4	5	6	7 Strongly Agree

There were positive relationships among the group members.

1 Strongly Disagree	2	3	4	5	6	7 Strongly Agree

Group members had a positive attitude toward the group leader.

1 7 Strongly Disagree	2	3	4	5	6

Group members participated actively in the group.

1 Strongly Disagree	2	3	4	5	6	7 Strongly Agree

How many participants actively participated? ______

Interventionist Characteristics

Group leader came prepared and organized.

1 Strongly Disagree	2	3	4	5	6	7 Strongly Agree

Group leader elicited clarification of participant engagement by seeking feedback about didactic content.

1 Strongly Disagree	2	3	4	5	6	7 Strongly Agree

Group leader delivered didactic material in an engaging, matter of fact, and respectful way.

1 Strongly Disagree	2	3	4	5	6	7 Strongly Agree

Group leader supplied the necessary materials for the participants.

1 Strongly Disagree	2	3	4	5	6	7 Strongly Agree

Group leader allocated time appropriately in order to cover the appropriate content focus points for the session.

1 Strongly Disagree	2	3	4	5	6	7 Strongly Agree

Group leader addressed process (tangential) issues but did not allow them to disrupt content agenda.

1	2	3	4	5	6	7
Strongly Disagree						Strongly Agree

Group leader avoided delving too deeply into psychological issues.

1	2	3	4	5	6	7
Strongly Disagree						Strongly Agree

Group leader responded empathically and accurately to participant behavior (verbal, nonverbal).

1	2	3	4	5	6	7
Strongly Disagree						Strongly Agree

Group leader facilitated discussion and interaction using open-ended questions, affirmations, reflections, and summaries.

1	2	3	4	5	6	7
Strongly Disagree						Strongly Agree

VA DPP Fidelity Checklist

Session Twelve: Ways to Stay Motivated

Site:____________________	Cohort:________________________	Group Leader:_________________
Evaluator: _______________	Attendance:____________________	Date:________________________
Scheduled Session Begin Time: __________________	Scheduled Session End Time: __________________	
Actual Time Session Began:_____________________	Actual Time Session Ended:____________________	

Instructions:

1. Review the session in the GLB workbook and the fidelity checklist prior to the session.

2. Complete *Part One: Food Dairies* before Session 12 begins based on the coach's comments in the food diaries collected in Session 11. *We want to capture the overall tone of the of the coach's comments; therefore a smiley face ☺ can be counted as a positive comment.*

3. Complete *Part Two: Session Content* during the session. Session activities were fully completed, partially completed, or not completed. **Use the comment section to explain the rating as needed, especially when an activity was partially completed (i.e. what aspect was complete, what aspect was incomplete). Please fill in each box in the table, including boxes next to sub questions.**

   Fully Completed Activities: this activity was fully completed with all participants

   Partially Completed Activities: this activity occurred, but did not occur with all group members or all of the time (i.e. group leader facilitated the discussion but only among certain members of the group or s/he began to demonstrate with an individual participant but did not follow through to adequate resolution)

   Not Completed Activities: this activity did not occur with any participants

4. Complete *Part Three: Session Principles* after the session based on the content checklist and your personal experience in the session. You will be assessing:

   Delivery of Educational Content
   Review of Goal Progress
   Goal Setting
   Group Cohesion
   Interventionist Characteristics

VA DPP Study Protocol Additional File 4: Session Delivery Fidelity Checklists for VA DPP and VA MOVE!

Part One: Food Dairies
Number of participants that attended Session 11.
____

Number of food diaries turned in at Session 11.
____

The group leader writes in green pen in:

- ☐ None of the diaries
- ☐ A few of the diaries (about ¼ of the diaries)
- ☐ About ½ of the diaries
- ☐ Most of the diaries (¾ of the diaries)
- ☐ All of the diaries

The group leader uses the *sandwich method* (one positive comment, one suggestion, and one positive comment) to comment on the front of:

- ☐ None of the diaries
- ☐ A few of the diaries (about ¼ of the diaries)
- ☐ About ½ of the diaries
- ☐ Most of the diaries (¾ of the diaries)
- ☐ All of the diaries

Inside each food diary, the group leader **generally** writes ____ positive comments or notations, i.e., a smiley face.
[Please note in the margin if there is an outlier to the general trend, i.e. most diaries have 4-6 comments but one diary didn't have any comments.]

- ☐ 0
- ☐ 1-3
- ☐ 4-6
- ☐ 7 or more

Inside each food diary, the group leader **generally** writes ____ suggestions for improvement.
[Please note in the margin if there is an outlier to the general trend, i.e. most diaries have 4-6 suggestions but one diary didn't have any suggestions.]

- ☐ 0
- ☐ 1-3
- ☐ 4-6
- ☐ 7 or more

Part Two: Session Content

Principle	Session Content	Checklist<br>0 = not completed<br>1 = partially completed<br>2 = fully completed	Comments
**Process**	Group leader elicits a report of any study or non-study related medical events from the group.		
**Goal Setting: Reviewing Progress**	Group leader weighs each patient privately and updates logs and graphs.<br>▪ If the patient has lost weight: Group leader congratulates participant, but stresses the fact that he or she must already be making some changes in behavior.<br>▪ If the participant has not lost weight: Group leader mentions it but stresses that little by little the numbers will change as the participant makes behavior changes.		
	Group leader asks if the participants weighed themselves at home; mentions that home weights and weights taken at the session may differ because scales differ, but the trend in weight change over time should be the same.		
**Process**	Group leader collects Keeping Track booklets and distributes blank booklets and booklets with comments to participants.		
**Process**	Group leader distributes session handouts.		

**Goal Setting: Reviewing Progress**	Group leader reviews the last session, including home activities, and the group discusses successes and difficulties in meeting the program goals.<br>▪ Did you have any trouble keeping track since the last session?<br>▪ Were you able to stay under your fat gram and calorie budgets?<br>▪ Were you able to follow your plan for physical activity, including lifestyle activity?<br>▪ Group leader reminds participants to graph their activity if they have not yet done so.<br>▪ The last session was "Make Social Cues Work for You." Did you follow your actions plans for changing a problem social cue and adding a positive social cue? What went well? What could you do differently?		
	▪ It's very important to keep in mind that, even though you have completed the initial 12 weekly sessions, the weight loss and physical activity goals are meant to last a lifetime.		
**Goal Setting: Goal Commitment**	Group leader emphasizes that participants who haven't reached their goals are to continue to strive toward reaching and maintaining them. Participants who have reached both goals are to strive to maintain them and surpass them if possible because they are minimum goals.		
**Goal Setting: Reviewing Progress**	Group leader asks the participants to review their progress since Session 1, and if not at goal, develop a plan to improve progress.<br>▪ What are some of the major changes you've made to be more active? Include both what you do to reach your goal (that is, those activities you record) and what you do to be more active in general (the		

	lifestyle activity that you don't record, like taking the stairs instead of an elevator).<br>▪ What changes have you made to eat fewer calories and less fat?		
	Group leader asks participants to record on the worksheet some of the changes they've made and instructs them to be as specific as possible. Group leader asks volunteers to share and praises and encourages the maintenance of these changes.<br>▪ Have you reached your weight goal? Your activity goal?		
	Group leader asks participants to refer to their How Am I Doing? graphs for weight and activity, and check yes or no on the worksheet. Group leader praises the progress made, both for participants at goal and those not at goal and encourages those not at goal to improve, and develop a plan using the worksheet. Some participants may need to be given meal plans at a lower calorie level.		
**Skill Development: Education**	Group leader discusses the importance of motivation.<br>▪ Motivation is crucial to maintaining healthy eating and physical activity for the long term; but how to stay motivated is one of the biggest problems people face. It is normal for your healthy lifestyle motivation to ebb and flow.		
	▪ Progress itself makes it hard to maintain that progress. Group leader tailors examples to participants regarding changes they've experienced that decrease their motivation; i.e. when you first came into the program, your clothes may have been tight and that motivated you to lose weight. If your clothes are looser on you now, you no longer have tight-fitting clothes as a source of motivation.		

	Group leader discusses ways to stay motivated.<br>**1. Stay aware of the benefits you've achieved and hope to achieve.**<br>▪ Think back to when you first joined the program. What did you hope to achieve?<br>Group leader asks participants to record their answers on the worksheet and refers the participants back to the worksheet from Session 1A, "Remember Your Purpose," and asks them to review their answers.<br>Group leader asks volunteers to share and acknowledges any costs that the participants articulate at this point (or that the participants have discussed before). Group leader acknowledges that everyone weighs the costs and benefits of healthy eating and staying active.<br>▪ Have you reached these goals?<br>▪ Have you received any benefits that you didn't expect?<br>Group leader asks participants what they would like to achieve during the next six months and asks them to make a list to review when they need motivation.<br>Group leader gives participants time to record their answers on the worksheet and asks volunteers to share.<br>**2. Recognize your successes.**<br>▪ What changes in your eating and activity habits do you feel proudest of?<br>▪ What has been easier than you thought it would be?<br>▪ What has been harder than you thought it would be?<br>▪ When you are feeling low on motivation, think about all of these positive changes and give yourself credit for them. Try not to lose the momentum you have		

	reached so far.<br>Group leader presents the "How Do Successful Weight Losers Do It?" handout if time permits.<br>▪ There is an old saying, "A path is made by walking on it." Researchers have studied people who've lost weight and kept it off. What steps did they take?<br>Group leader reviews the "before and after" steps on the handout with the group, then asks participants to reflect on and answer the two questions at the bottom of the page and asks volunteers to share.<br>**3. Keep visible signs of your progress so you can see how far you've come.**<br>▪ Post a graph of your weight loss and activity on your refrigerator door. Not only will it keep you aware of your progress, but loved ones will take note and congratulate you for your movement in the right direction.<br>▪ Mark your activity milestones on a map toward a particular goal. For example, create a simple map of the number of miles it would take to walk to a favorite vacation spot or tour a favorite city. Mark milestones along the way (the halfway point, a fun museum to stop at along the way, and so on). You might even want to go on an actual vacation at that place when you reach your goal.<br>4. Measure yourself at monthly intervals. Keep track of your progress in terms of specific measurements (for example, waist circumference or the number of belt loops).**Keep track of your weight, eating and activity**.<br>▪ It's common to "drift" away from new habits. You		

	may gradually make small changes in your eating and activity over a long period of time, and not even be aware that you are slowly going back to your old habits. The best way to prevent this and stay in control is to continue to keep track. Keeping track will help you catch changes before they sneak up on you.<br>Group leader gives the participants optional forms for self-monitoring during maintenance, such as the Group Lifestyle Balance Calendar, if applicable.<br>▪ Record your activity daily.<br>▪ Record what you eat this often:___________.<br>▪ Record your weight on___________.<br>▪ If you gain weight, you will need to keep track more often.<br>**5. Consider whether you need to add variety to your routine.**<br>Group leader discusses activity and variety: We've talked before about how to "jump start" your activity plan.<br>▪ Have you added some variety to keep yourself from being bored with staying active?<br>▪ Have you noticed any difference in how you feel about being active?<br>Group leader discusses diet and variety: The same thing may be true with eating. If you have grown tired of using the same low fat salad dressing every night, experiment with some new products. Consider trying new recipes and restaurants. If you are the kind of person who has a desire to be more creative there are many magazines and websites that provide an outlet to explore new, healthy meals. On the other hand, if you are the kind of person		

	who is comfortable with "routine eating" and simply do not require that much variation in your breakfast, lunch and dinner, that is perfectly fine too. Some people report doing better, over time, with structured meals and minimal variety, others do not. What is important is to get to know who you are and plan accordingly.<br>▪ What meals, snacks, or particular foods are you most bored with? Can you think of some ways to vary this part of your eating?<br>Group leader asks participants to share their ideas.<br>▪ If participants express interest in learning more about a specific topic such as ethnic cooking or vegetarian eating, group leader addresses it briefly and if possible directs them to where to find books and/or courses on these topics.<br>**6. Manage Stress**<br>Stress is tension or pressure. Stress is a natural part of living our life. Any change, good or bad, big or small, can cause stress. Big changes or events in our life – like getting married, a serious illness, changing jobs – can cause stress. Small events – like losing your keys, having a birthday, having a flat tire, or needing to get your errands done before picking up your children – can also cause stress.<br>▪ What kinds of things make you feel stressed?<br>Group leader gives participants time to record their answers and asks volunteers to share.<br>▪ Many people react to stress by changing their eating and activity habits. Some people eat and drink too much as a way to deal with stress. Others may stop		

	eating. Some people become very inactive and withdrawn.<br>▪ What is it like for you when you get stressed?<br>Group leader mentions one or two situations that are typically stressful and asks how the participants feel or react in such situations.<br>Group leader gives participants time to record their answers and asks volunteers to share.<br>Based on the responses, group leader points out any physical and emotional symptoms (headache or muscle tension) and behavioral changes that might affect eating and activity.<br>▪ Do you get any physical symptoms like a headache or stomach ache or muscle tension?<br>▪ Do you change your behaviors when you feel stressed?<br>▪ Do you eat more when you are stressed?<br>▪ Do you change the kinds of food you eat?<br>▪ Do you change how active you are or the kind of physical activities you do?<br>Group leader states that the best approach is to prevent stress whenever you can:<br>▪ Practice saying, "No."<br>▪ Share some of your work with others, both at home and at work: delegate what you can to someone else.<br>▪ Set goals you can reach.<br>▪ Take charge of your time: make schedules with the real world in mind and get organized.<br>▪ Use the steps for solving problems.<br>▪ Plan ahead.		

	▪ Keep things in perspective and remember your purpose.<br>▪ Reach out to people.<br>▪ Be physically active.<br>▪ What about times when you can't avoid stress?<br>Group leader discuses action or behavior chains and the importance of trying to break them as early as possible. Group leader explains the same is true of stress; if you learn to recognize the signs of stress and catch yourself early in the process, you may have a chance to avoid some of the harmful consequences such as overeating or being inactive.<br>▪ Do you have any signs when you are getting stressed?<br>Group leader encourages participants to take a 10-minute "time out." Develop a new habit of responding to stress with a "time out" – stop what you are doing and take a few minutes for yourself. Do whatever you find helpful that doesn't involve food.<br>▪ Move those muscles.<br>▪ Pamper yourself.<br>▪ Breathe.<br>Group leader states that lifestyle changes may cause stress and reviews the "When you Can't Avoid Stress" worksheet with participants. Group leader notes that some of the possible sources of stress may not apply to every participant.<br>Group leader does not allow review of the worksheet to create a negative perspective, and helps the participants feel able to cope should such stresses arise.<br>7. **Set new goals for yourself, and develop ways to reward yourself when you meet each goal.**<br>▪ The goal should be specific, short-term and not too easy or too hard.		

	▪ The reward should be something that you will do or buy if and only if you reach your goal.<br>▪ What are some non-food ways you can reward yourself for reaching a goal?<br>Group leader asks participants to record their ideas on the worksheet and asks volunteers to share.<br>Group leader presents "What's Your Pleasure?" and "Let Me Count the Ways" worksheets if time permits.<br>▪ When you feel discouraged, remember, it takes time to change. You deserve to be good to yourself for all of the efforts you make. What are three things you enjoy doing but rarely do? Choose things that aren't related to food and that are within the realm of possibility.<br>8. **Consider creating some friendly competition.**<br>▪ Get a friend or relative to enter into a friendly competition with you. This should be the kind of competition in which you both win.<br>9. **Use others to help you stay motivated.**<br>▪ If you notice that your motivation is dropping, talk with someone who is supportive. Everyone has trouble staying motivated sometimes, and we can encourage each other through the tough times.		
**Goal Setting: Goal Commitment**	Group leader asks participants to make an action plan related to staying motivated.<br>▪ Pick one way to stay motivated that you think would be most helpful to you right now. Choose something that is very likely to work and that you can do. Be realistic. Be specific. Then make a positive action plan.<br>Group leader gives participants time to record their plan on the worksheet.		

	Group leader offers congratulations on completing the first 12 sessions of the Diabetes Prevention Program and tells participants they deserve credit for the changes they have made, no matter how small. It's a challenge to change eating and activity habits, and every step along the way is a reason to celebrate.		
	Group leader asks participants to set goals for the next phase of the program.<br>▪ Now we are beginning the transition to the next phase of the program. Our sessions will be less frequent and it is important to consider your longer term goals for activity, weight, self-monitoring and social support for staying on track.<br>▪ First, you will need to keep in mind why reaching and staying at a healthy weight and being active are important to you.<br>Group leader gives participants time to answer this and the remaining questions on the "My Personal Lifestyle Contract" worksheet.<br>Group leader encourages participants to continue to strive to reach and maintain at least 150 minutes per week of moderate physical activity and weigh themselves at least once a week.<br>Group leader stresses the importance of having a specific plan for weight regain of a specific amount.		
**Process**	Group leader distributes the "Group Lifestyle Balance Calendar" as an option for self-monitoring as well as any other weight, diet, or activity tracking sheets they have developed for the participant notebooks and any handouts they've developed on sources of support for participants after the program, such as how to contact a registered dietitian, where to be active in the community, or courses on healthy eating and activity topics.		
**Goal Setting:**	Group leader has participants complete the "To Do"		

**Goal Commitment**	worksheet if they have not already done so during the session.		
**Process**	Group leader asks participants if they have any questions and tells them that their next group meeting will be in two weeks.		
	Group leader reviews the schedule of classes for the remainder of the year and emphasizes the importance of continuing to attend the VA DPP support sessions.		
	Group leader congratulates participants again for completing this portion of the program and tells them that they have gained important self-management strategies that will serve them well.		
	Group leader reminds them that their motivation may ebb and flow, but the learning tools will help them each time they pick them up.		
	Group leader informs them that as the group moves into the next part of the program, they will be revisiting some strategies and learning new ones to help keep everyone on track over the long run.		
	Group leader tells everyone s/he is looking forward to seeing everyone soon!		
**What activities or additional topics were added to any aspect of the session?**			

**Additional comments about the session?**	

Part Three: Session Principles

Delivery of Educational Content
Group leader elicited discussion of the educational content in order to help participants develop a self-management skill or change cognitions.

Group leader delivered approximately ______% percent of the educational content to the participants.

Review of Goal Progress
Group leader prompted review of goal progress and attainment.

1<br>Strongly Disagree	2	3	4	5	6	7<br>Strongly Agree

Group leader elicited discussion of successes and challenges since the last session.

1<br>Strongly Disagree	2	3	4	5	6	7<br>Strongly Agree

Group leader initiated problem solving when necessary to address challenges since the last session.

1<br>Strongly Disagree	2	3	4	5	6	7<br>Strongly Agree

Goal Setting
Diabetes prevention was discussed as a goal of the group.

1<br>Strongly Disagree	2	3	4	5	6	7<br>Strongly Agree

Group leader presented standardized goals (i.e. everyone had the same goal to complete the following week) to the participants and asked them to commit to a goal.

1<br>Strongly Disagree	2	3	4	5	6	7<br>Strongly Agree

Group Cohesion
Group identity includes having a diagnosis of prediabetes.
[*Coach and/or group members acknowledge a commonality among members in being diagnosed with prediabetes.*]

1<br>Strongly Disagree	2	3	4	5	6	7<br>Strongly Agree

There were new members present in the session.
☐ No
☐ Yes

Group members communicated easily with one another.

1 Strongly Disagree	2	3	4	5	6	7 Strongly Agree

There were positive relationships among the group members.

1 Strongly Disagree	2	3	4	5	6	7 Strongly Agree

Group members had a positive attitude toward the group leader.

1	2	3	4	5	6
7					
Strongly Disagree					

Group members participated actively in the group.

1 Strongly Disagree	2	3	4	5	6	7 Strongly Agree

How many participants actively participated? ______

Interventionist Characteristics

Group leader came prepared and organized.

1 Strongly Disagree	2	3	4	5	6	7 Strongly Agree

Group leader elicited clarification of participant engagement by seeking feedback about didactic content.

1 Strongly Disagree	2	3	4	5	6	7 Strongly Agree

Group leader delivered didactic material in an engaging, matter of fact, and respectful way.

1 Strongly Disagree	2	3	4	5	6	7 Strongly Agree

Group leader supplied the necessary materials for the participants.

1 Strongly Disagree	2	3	4	5	6	7 Strongly Agree

Group leader allocated time appropriately in order to cover the appropriate content focus points for the session.

1 Strongly Disagree	2	3	4	5	6	7 Strongly Agree

VA DPP Fidelity Checklist

MOVE! Session

Site:__________________________________	MOVE! Teacher:____________________________________
Evaluator: _______________ Attendance:____________________	Date:________________________
Scheduled Session Begin Time: __________________	Scheduled Session End Time: __________________
Actual Time Session Began:_____________________	Actual Time Session Ended:____________________

Instructions:

1. Review the fidelity checklist prior to the session.

2. If the MOVE! coordinator regularly collects food diaries, complete *Part One: Food Dairies* before the session begins based on the coach's comments in the food diaries collected in the previous session. *We want to capture the overall tone of the of the coach's comments; therefore a smiley face ☺ can be counted as a positive comment.* If the MOVE! coordinator does not regularly collect food diaries, please note this in the margin in part one.

3. Complete *Part Two: Session Content* during the session. Session activities were fully completed, partially completed, or not completed. **Use the comment section to explain the rating as needed, especially when an activity was partially completed (i.e. what aspect was complete, what aspect was incomplete). Please fill in each box in the table, including boxes next to sub questions.**

   Fully Completed Activities: this activity was fully completed with all participants

   Partially Completed Activities: this activity occurred, but did not occur with all group members or all of the time (i.e. group leader facilitated the discussion but only among certain members of the group or s/he began to demonstrate with an individual participant but did not follow through to adequate resolution)

   Not Completed Activities: this activity did not occur with any participants

4. Complete *Part Three: Session Principles* after the session based on the content checklist and your personal experience in the session. You will be assessing:

   Delivery of Educational Content
   Review of Goal Progress
   Goal Setting
   Group Cohesion
   Interventionist Characteristics

VA DPP Study Protocol Additional File 4: Session Delivery Fidelity Checklists for VA DPP and VA MOVE!

Part One: Food Dairies
Number of participants that attended previous session.
____

Number of food diaries turned in at previous session.
____

The group leader writes in green pen in:

- ☐ None of the diaries
- ☐ A few of the diaries (about ¼ of the diaries)
- ☐ About ½ of the diaries
- ☐ Most of the diaries (¾ of the diaries)
- ☐ All of the diaries

The group leader uses the *sandwich method* (one positive comment, one suggestion, and one positive comment) to comment on the front of:

- ☐ None of the diaries
- ☐ A few of the diaries (about ¼ of the diaries)
- ☐ About ½ of the diaries
- ☐ Most of the diaries (¾ of the diaries)
- ☐ All of the diaries

Inside each food diary, the group leader **generally** writes ____ positive comments or notations, i.e., a smiley face.
[Please note in the margin if there is an outlier to the general trend, i.e. most diaries have 4-6 comments but one diary didn't have any comments.]

- ☐ 0
- ☐ 1-3
- ☐ 4-6
- ☐ 7 or more

Inside each food diary, the group leader **generally** writes ____ suggestions for improvement.
[Please note in the margin if there is an outlier to the general trend, i.e. most diaries have 4-6 suggestions but one diary didn't have any suggestions.]

- ☐ 0
- ☐ 1-3
- ☐ 4-6
- ☐ 7 or more

Part Two: Session Content

**Principle**	**Session Content**	**Checklist**<br>0 = not completed<br>1 = partially completed<br>2 = fully completed	**Comments**
**Process**	Group leader handed out all of the session materials.		Please attach the session materials to the fidelity checklist. If it is not possible to receive the handouts, please note the number of handouts.
**Goal Setting: Reviewing Progress**	Group reviewed member's progress in reaching goals.		Describe how this occurred.

**Principle**	**Session Content**	**Checklist**<br>0 = not completed<br>1 = partially completed<br>2 = fully completed	**Comments**
**Skill Development: Problem Solving**	Group leader helped participants solve problems to overcome challenges in meeting their goals.		Describe how this occurred.
**Goal Setting: Goal Commitment**	Group set new goals for the following week.		What goals did the group set? Did everyone set the same goal?

Principle	Session Content	Checklist<br>0 = not completed<br>1 = partially completed<br>2 = fully completed	Comments
**Skill Development: Education**	Group learned about the assigned topic.		Please list the topics that were covered.
**Additional comments about the session?**			

Part Three: Session Principles

Delivery of Educational Content
Group leader elicited discussion of the educational content in order to help participants develop a self-management skill or change cognitions.

Group leader delivered approximately ______% percent of the educational content to the participants.

Review of Goal Progress
Group leader prompted review of goal progress and attainment.

1	2	3	4	5	6	7
Strongly Disagree						Strongly Agree

Group leader elicited discussion of successes and challenges since the last session.

1	2	3	4	5	6	7
Strongly Disagree						Strongly Agree

Group leader initiated problem solving when necessary to address challenges since the last session.

1	2	3	4	5	6	7
Strongly Disagree						Strongly Agree

Goal Setting
Diabetes prevention was discussed as a goal of the group.

1	2	3	4	5	6	7
Strongly Disagree						Strongly Agree

Group leader presented standardized goals (i.e. everyone had the same goal to complete the following week) to the participants and asked them to commit to a goal.

1	2	3	4	5	6	7
Strongly Disagree						Strongly Agree

Group Cohesion
Group identity includes having a diagnosis of prediabetes.
[*Coach and/or group members acknowledge a commonality among members in being diagnosed with prediabetes.]*

1	2	3	4	5	6	7
Strongly Disagree						Strongly Agree

There were new members present in the session.
☐ No
☐ Yes

Group members communicated easily with one another.

1	2	3	4	5	6	7
Strongly Disagree						Strongly Agree

There were positive relationships among the group members.

1	2	3	4	5	6	7
Strongly Disagree						Strongly Agree

Group members had a positive attitude toward the group leader.

1	2	3	4	5	6	7
Strongly Disagree						

Group members participated actively in the group.

1	2	3	4	5	6	7
Strongly Disagree						Strongly Agree

How many participants actively participated? ______

Interventionist Characteristics

Group leader came prepared and organized.

1	2	3	4	5	6	7
Strongly Disagree						Strongly Agree

Group leader elicited clarification of participant engagement by seeking feedback about didactic content.

1	2	3	4	5	6	7
Strongly Disagree						Strongly Agree

Group leader delivered didactic material in an engaging, matter of fact, and respectful way.

1	2	3	4	5	6	7
Strongly Disagree						Strongly Agree

Group leader supplied the necessary materials for the participants.

1	2	3	4	5	6	7
Strongly Disagree						Strongly Agree

Group leader allocated time appropriately in order to cover the appropriate content focus points for the session.

1	2	3	4	5	6	7
Strongly Disagree						Strongly Agree
