# Supplementary material for: Implementation and evaluation of the VA DPP clinical demonstration: protocol for a multi-site non-randomized hybrid effectiveness-implementation type III trial
Source: Implement Sci. 2015 May 12;10:68. doi: 10.1186/s13012-015-0250-0 (PMC4429938; doi:10.1186/s13012-015-0250-0)
Supplement: Additional file 5: — Program and EuroQOL EQ-5D-5 L surveys. This file provides a copy of the program and EuroQOL EQ-5D-5 L surveys. [file 13012_2015_250_MOESM5_ESM.pdf]

Study ID: __ __ __ __ __ __

DIABETES PREVENTION SURVEY

Program Follow-Up Survey

SURVEY COMPLETION DATE:

____ / ____ / ______
Month Day Year

Thank you for being a part of our study! The information you provide on this survey will help us keep track of how you feel and how well you are able to do your usual activities. If you have any questions, please contact Bradley Youles at 1-800-753-3357.

Please answer each question by marking the answer as instructed. If you are unsure how to answer a question, please give the best answer you can. Unless otherwise indicated, please choose only one answer for each question.

Participation

1. Which program did you participate in?

☐1 In-Person Group VA DPP
☐2 Online VA DPP
☐3 MOVE!
☐4 None, I did not participate in any of the programs

General Health

2. In general, would you say that your health is:

☐1 Poor
☐2 Fair
☐3 Good
☐4 Very good
☐5 Excellent

3. **The following questions are about activities you might do during a typical day. Does your health now limit you in these activities? If so, how much?**

	Yes, limited a lot [1]	Yes, limited a little [2]	No, not limited at all [3]
a. **Moderate activities**, such as moving a table, pushing a vacuum cleaner, bowling, or playing golf	☐	☐	☐
b. Climbing **several** flights of stairs	☐	☐	☐

4. **During the past 4 weeks, have you had any of the following problems with your work or other regular daily activities as a result of your physical health?**

	No, none of the	Yes, a little of	Yes, some of the	Yes, most of the	Yes, all of the time

	time [1]	the time [2]	time [3]	time [4]	[5]
a. **Accomplished less** than you would like	☐	☐	☐	☐	☐
b. Were limited in the **kind** of work or other activities	☐	☐	☐	☐	☐

5. **During the past 4 weeks, have you had any of the following problems with your work or other regular daily activities as a result of any emotional problems (such as feeling depressed or anxious)?**

	No, none of the time [1]	Yes, a little of the time [2]	Yes, some of the time [3]	Yes, most of the time [4]	Yes, all of the time [5]
a. **Accomplished less** than you would like	☐	☐	☐	☐	☐
b. Were limited in the **kind** of work or other activities	☐	☐	☐	☐	☐

6. During the past 4 weeks, how much did **pain** interfere with your normal work (including both work outside the home and housework)?

☐1 Not at all
☐2 A little bit
☐3 Moderately
☐4 Quite a bit
☐5 Extremely

7. **These next questions are about how you feel and how things have been with you during the past 4 weeks. For each question, please give the one answer that comes closest to the way you have been feeling.**

**How much of the time during the past 4 weeks:**	All of the time [1]	Most of the time [2]	A good bit of the time [3]	Some of the time [4]	A little of the time [5]	None of the time [6]
a. Have you felt **calm and peaceful**?	☐	☐	☐	☐	☐	☐
b. Did you have **a lot of energy**?	☐	☐	☐	☐	☐	☐
c. Have you felt **downhearted and blue**?	☐	☐	☐	☐	☐	☐

8. During the past 4 weeks, how much of the time has your **physical or emotional problems** interfered with your social activities (like visiting friends, relatives, etc.)?

☐1 All of the time
☐2 Most of the time
☐3 Some of the time
☐4 A little of the time
☐5 None of the time

Now, we'd like to ask you some questions about how your health may have changed.

9. Compared to 1 year ago, how would you rate your **physical health** in general **now**?

☐1 Much better
☐2 Slightly better
☐3 About the same
☐4 Slightly worse
☐5 Much worse

10. Compared to 1 year ago, how would you rate your **emotional problems** (such as feeling anxious, depressed or irritable) **now**?

☐1 Much better
☐2 Slightly better
☐3 About the same
☐4 Slightly worse
☐5 Much worse

Diet Behaviors

11. How many servings of fruits do you eat every day? (Examples of a serving size include a medium-sized apple, a ½ cup of grapes, or a ¼ cup of raisins.)

- ☐1 None
- ☐2 1-2
- ☐3 3-4
- ☐4 5-6
- ☐5 7 or more

12. How many servings of vegetables do you eat every day? (Examples of a serving size include a medium-sized potato, a cup of lettuce, or a ½ cup of beans.)

- ☐1 None
- ☐2 1-2
- ☐3 3-4
- ☐4 5-6
- ☐5 7 or more

13. How often do you eat sugary foods?

- ☐1 Never
- ☐2 Once a month or less
- ☐3 Once a week
- ☐4 At least 3 times a week
- ☐5 Every day

14. Check the box that describes how much you agree with each of the following statements:

	Definitely true [0]	Mostly true [1]	Mostly false [2]	Definitely false [3]
a. When I smell a sizzling steak or juicy piece of meat, I find it very difficult to keep from eating, even if I have just finished a meal.	☐	☐	☐	☐
b. I deliberately take small helpings as a means of controlling my weight.	☐	☐	☐	☐
c. When I feel anxious, I find myself eating.	☐	☐	☐	☐
d. Sometimes when I start eating, I just can't seem to stop.	☐	☐	☐	☐
e. Being with someone who is eating often makes me hungry enough to eat also.	☐	☐	☐	☐
f. When I feel blue, I often overeat.	☐	☐	☐	☐
g. When I see a real delicacy, I often get so hungry that I have to eat right away.	☐	☐	☐	☐
h. I get so hungry that my stomach often seems like a bottomless pit.	☐	☐	☐	☐
i. I am always hungry so it is hard for me to stop eating before I finish the food on my plate.	☐	☐	☐	☐
j. When I feel lonely, I console myself by eating.	☐	☐	☐	☐
k. I consciously hold back at meals in order not to gain weight.	☐	☐	☐	☐
l. I do not eat some foods because they make me fat.	☐	☐	☐	☐
m. I am always hungry enough to eat at any time.	☐	☐	☐	☐

15. How often do you feel hungry?

- ☐1 Only at meal times
- ☐2 Sometimes between meals
- ☐3 Often between meals
- ☐4 Almost always

16. How frequently do you **avoid** "stocking up" on tempting foods?

- ☐1 Almost never
- ☐2 Seldom
- ☐3 Usually
- ☐4 Almost always

17. How likely are you to consciously eat less than you want?

- ☐1 Unlikely
- ☐2 Slightly unlikely
- ☐3 Moderately likely
- ☐4 Likely

18. Do you go on eating binges though you are not hungry?

- ☐1 Never
- ☐2 Rarely
- ☐3 Sometimes
- ☐4 At least once a week

19. On a scale of 1 to 8, where 1 means no restraint in eating (eating whatever you want, whenever you want it) and 8 means total restraint (constantly limiting food intake and never "giving in"), what number would you give yourself?

Please circle one number.

1	2	3	4	5	6	7	8
No restraint in eating							Total restraint in eating

<u>**Exercise Behaviors**</u>

The next set of questions asks about your physical activity habits. There are two types of activities to consider:

<u>**Moderate activities**</u> cause light sweating and a slight to moderate increase in breathing or heart rate. Examples include brisk walking, bicycling, vacuuming, gardening, and golfing without a cart.

<u>**Vigorous activities**</u> cause heavy sweating and large increases in breathing or heart rate. Examples include running, aerobic classes, heavy yard work, and briskly swimming laps.

20. How many days per week do you do **moderate activities** for at least 10 minutes at a time?

Please circle <u>one</u> number.

0	1	2	3	4	5	6	7

21. On days when you do **moderate activities** for at least 10 minutes at a time, how much total time per day do you spend doing these activities?

- ☐1 10-19 minutes
- ☐2 20-29 minutes
- ☐3 30-59 minutes
- ☐4 60 minutes or more
- ☐5 Not applicable, I circled "0" for the question above

22. How many days per week do you do **vigorous activities** for at least 10 minutes at a time?

Please circle <u>one</u> number.

0	1	2	3	4	5	6	7

23. On days when you **do vigorous activities** for at least 10 minutes at a time, how much total time per day do you spend doing these activities?

- ☐1 10-19 minutes
- ☐2 20-29 minutes
- ☐3 30-59 minutes
- ☐4 60 minutes or more
- ☐5 Not applicable, I circled "0" for the question above

Sleep Behaviors

24. Overall, how would you rate how much sleep you get on average?

- ☐1 Very good
- ☐2 Good
- ☐3 Fair
- ☐4 Poor
- ☐5 Very poor

25. Overall, how would you rate the quality of your sleep on average?

- ☐1 Very good
- ☐2 Good
- ☐3 Fair
- ☐4 Poor
- ☐5 Very poor

Diabetes Risk

The next set of questions asks about how you view your risk of getting diabetes.

26. What are your chances of getting Type 2 Diabetes in your lifetime?

Please circle one number.

1	2	3	4	5	6	7
Definitely will not get diabetes						Definitely will get diabetes

27. Please list, *in order of importance*, the three things you can do that might prevent you from getting diabetes.

1. ______________________________
2. ______________________________
3. ______________________________

Anticipated Benefit of Weight Loss

28. The major benefits of weight loss are:

	Strongly disagree [1]	Disagree [2]	Neutral [3]	Agree [4]	Strongly agree [5]
a. Staying in shape	☐	☐	☐	☐	☐
b. Making me feel better in general	☐	☐	☐	☐	☐
c. Good health	☐	☐	☐	☐	☐
d. Maintaining proper body weight	☐	☐	☐	☐	☐
e. Improving appearance	☐	☐	☐	☐	☐
f. Enhancing self-image and confidence	☐	☐	☐	☐	☐
g. Positive psychological effect	☐	☐	☐	☐	☐
h. Reducing stress and relax	☐	☐	☐	☐	☐
i. Fun and enjoyment	☐	☐	☐	☐	☐
j. Helping cope with life’s pressures	☐	☐	☐	☐	☐
k. Reducing pain	☐	☐	☐	☐	☐
l. Companionship	☐	☐	☐	☐	☐
m. Maintaining independence as I get older	☐	☐	☐	☐	☐
n. Setting a good example for my children/other children	☐	☐	☐	☐	☐

29. Are there any other benefits of weight loss that would be good for you?

____

____

Motivation

If you do not follow a diet plan or exercise regularly, please skip to question 31.

30. **The reason I follow my diet and exercise regularly is that:**

Please circle one number.

a. The reason I follow my diet and exercise regularly is that:
Other people would be upset with me if I didn't.

1	2	3	4	5	6	7
Not at all true						Very true

b. The reason I follow my diet and exercise regularly is that:
I personally believe that these are important in remaining healthy.

1	2	3	4	5	6	7
Not at all true						Very true

c. The reason I follow my diet and exercise regularly is that:
I would be ashamed of myself if I didn't.

1	2	3	4	5	6	7
Not at all true						Very true

d. The reason I follow my diet and exercise regularly is that:
It is easier to do what I'm told than to think about it.

1	2	3	4	5	6	7
Not at all true						Very true

e. The reason I follow my diet and exercise regularly is that:
I've carefully thought about my diet and exercising and believe it's the right thing to do.

1	2	3	4	5	6	7
Not at all true						Very true

f. The reason I follow my diet and exercise regularly is that:
I want others to see that I can follow my diet and stay fit.

1	2	3	4	5	6	7
Not at all true						Very true

g. The reason I follow my diet and exercise regularly is that:
I just do it because my doctor said to.

1	2	3	4	5	6	7
Not at all true						Very true

h. The reason I follow my diet and exercise regularly is that:
I feel personally that watching my diet and exercising are the best things for me.

1	2	3	4	5	6	7
Not at all true						Very true

i. The reason I follow my diet and exercise regularly is that:
I'd feel guilty if I didn't watch my diet and exercise.

1	2	3	4	5	6	7
Not at all true						Very true

j. The reason I follow my diet and exercise regularly is that:
Exercising regularly and following my diet are choices I really want to make.

1	2	3	4	5	6	7
Not at all true						Very true

31. **How motivated are you to:**

	Very motivated [1]	Somewhat motivated [2]	Neutral [3]	Somewhat unmotivated [4]	Not motivated at all [5]
a. Lose weight	☐	☐	☐	☐	☐
b. Exercise	☐	☐	☐	☐	☐
c. Eat a healthy diet	☐	☐	☐	☐	☐
d. Avoid getting diabetes	☐	☐	☐	☐	☐

Goal Awareness and Commitment - Diet, Physical Activity, and Weight

32. Did you set a daily calorie goal?

- ☐0 No → ***If you did not set a daily calorie goal, please skip to question 36.***
- ☐1 Yes

    a. If **Yes**, what was your daily calorie goal? ___________________________________

    b. If **Yes**, how did you determine your daily calorie goal?
    - ☐1 I chose this myself
    - ☐2 My group leader chose this goal
    - ☐3 I worked with my group leader to come up with this goal
    - ☐4 Other (please specify):____

33. Did you feel that the goals you set with the group leader helped you improve your eating habits?

- ☐1 Yes, very helpful
- ☐2 Yes, somewhat helpful
- ☐3 No, not helpful
- ☐4 Not applicable, I was not in a program

34. Did you have enough say about developing your goals for nutritional change?

- ☐1 Yes, definitely
- ☐2 Yes, somewhat
- ☐3 No
- ☐4 Not applicable, I was not in a program

35. Did you find keeping track of what you ate helpful to make goals related to changing your eating habits?

- ☐1 Yes, very helpful
- ☐2 Yes, somewhat helpful
- ☐3 No, not helpful
- ☐4 Not applicable, I did not keep track of what I ate

36. Did you set a daily physical activity goal?

- ☐0 No → ***If you did not set a physical activity goal, please skip to question 40.***
- ☐1 Yes

   a. If **Yes**, what was your daily physical activity goal? ____________________________

   b. If **Yes**, how did you determine your daily physical activity goal?
   - ☐1 I chose this myself
   - ☐2 My group leader chose this goal
   - ☐3 I worked with my group leader to come up with this goal
   - ☐4 Other (please specify):____

37. Did you feel that the goals you set with the group leader helped you improve your level of physical activity?

- ☐1 Yes, very helpful
- ☐2 Yes, somewhat helpful
- ☐3 No, not helpful
- ☐4 Not applicable, I was not in a program

38. Did you have enough say about developing your goals for physical activity?

- ☐1 Yes, definitely
- ☐2 Yes, somewhat
- ☐3 No
- ☐4 Not applicable, I was not in a program

39. Did you find keeping track of your physical activity helpful to make goals related to changing your physical activity?

- ☐1 Yes, very helpful
- ☐2 Yes, somewhat helpful
- ☐3 No, not helpful
- ☐4 Not applicable, I did not keep track of my physical activity

40. Did you set a weight loss goal?

- ☐ 0 No
- ☐ 1 Yes

  a. If **Yes**, what was your weight loss goal? ___________________________________

  b. If **Yes**, how did you determine your weight loss goal?
  - ☐ 1 I chose this myself
  - ☐ 2 My group leader chose this goal
  - ☐ 3 I worked with my group leader to come up with this goal
  - ☐ 4 Other (please specify):____

41. **Please select the <u>one</u> answer that best matches how much you agree with the following statements.** ***If you did not set any goals, please skip to question 42.***

	Strongly agree [1]	Agree [2]	Neither agree nor disagree [3]	Disagree [4]	Strongly disagree [5]
a. I am very committed to my daily calorie goal.	☐	☐	☐	☐	☐
b. I am very committed to my daily physical activity goal.	☐	☐	☐	☐	☐
c. I am very committed to my weight loss goal.	☐	☐	☐	☐	☐
d. I am very confident I will meet my daily calorie goal.	☐	☐	☐	☐	☐
e. I am very confident I will meet my daily physical activity goal.	☐	☐	☐	☐	☐
f. I am very confident I will meet my weight loss goal.	☐	☐	☐	☐	☐

Social Support – Exercise

42. **Below is a list of things people might do or say to someone who is trying to exercise regularly. Please rate how often anyone has said or done what is described during the past 3 months.**

**During the past 3 months, my family (or members of my household), friends, or online friends/support groups:**	None [1]	Rarely [2]	A Few Times [3]	Often [4]	Very Often [5]
a. Exercised with me.	☐	☐	☐	☐	☐
b. Offered to exercise with me.	☐	☐	☐	☐	☐
c. Gave me helpful reminders to exercise.	☐	☐	☐	☐	☐
d. Gave me encouragement to stick with my exercise program.	☐	☐	☐	☐	☐
e. Changed their schedule so we could exercise together.	☐	☐	☐	☐	☐
f. Discussed exercise with me.	☐	☐	☐	☐	☐
g. Complained about the time I spend exercising.	☐	☐	☐	☐	☐
h. Criticized me or made fun of me for exercising.	☐	☐	☐	☐	☐
i. Gave me rewards for exercising.	☐	☐	☐	☐	☐
j. Planned for exercise on recreational outings.	☐	☐	☐	☐	☐
k. Helped plan activities around my exercise.	☐	☐	☐	☐	☐
l. Asked me for ideas on how they can get more exercise.	☐	☐	☐	☐	☐
m. Talked about how much they like to exercise.	☐	☐	☐	☐	☐

Social Support – Diet

43. **Below is a list of things people might do or say to someone who is trying to eat a healthy diet. Please rate how often anyone has said or done what is described during the past 3 months.**

**During the past 3 months, my family (or members of my household), friends, or online friends/support groups:**	None [1]	Rarely [2]	A Few Times [3]	Often [4]	Very Often [5]
a. Ate healthy foods with me.	☐	☐	☐	☐	☐
b. Offered to cook healthy foods with me.	☐	☐	☐	☐	☐
c. Gave me helpful reminders to eat well.	☐	☐	☐	☐	☐
d. Gave me encouragement to stick with my diet.	☐	☐	☐	☐	☐
e. Changed their food habits so we could eat together.	☐	☐	☐	☐	☐
f. Discussed healthy eating with me.	☐	☐	☐	☐	☐
g. Complained about the healthy food I eat.	☐	☐	☐	☐	☐
h. Criticized me or made fun of me for my healthy food choices.	☐	☐	☐	☐	☐
i. Gave me rewards for eating well.	☐	☐	☐	☐	☐
j. Planned ahead for healthy food decisions when going out to restaurants.	☐	☐	☐	☐	☐
k. Helped plan meals around my diet.	☐	☐	☐	☐	☐
l. Asked me for ideas on how they can eat better.	☐	☐	☐	☐	☐
m. Talked about how much they like to eat better.	☐	☐	☐	☐	☐

Confidence

44. How confident are you that you can maintain a physically active lifestyle for the next 12 months?

Please circle one number.

1	2	3	4	5
Not at all confident				Very confident

45. How confident are you that you can maintain a healthy diet for the next 12 months?

Please circle one number.

1	2	3	4	5
Not at all confident				Very confident

46. How confident are you that you can avoid gaining weight for the next 12 months?

Please circle one number.

1	2	3	4	5
Not at all confident				Very confident

Willingness to Self-Monitor

The following questions relate to how willing you are to participate in specific behaviors that contribute to successful weight loss and a healthy lifestyle.

47. Please indicate the number of times per week you are willing to do the following:

	**Circle the number indicating how many times per week you are willing to do the behavior**						
a. To write down everything I eat and drink.	1	2	3	4	5	6	7
b. To record the number of calories that I eat.	1	2	3	4	5	6	7
c. To record the amount of fat grams that I eat.	1	2	3	4	5	6	7
d. To measure my food portions using scales, spoons, cups, etc.	1	2	3	4	5	6	7
e. To purposely eat smaller portion sizes of food.	1	2	3	4	5	6	7
f. To substitute water for high calorie/sugar-filled beverages	1	2	3	4	5	6	7
g. To record the physical activity that I do (in minutes or steps).	1	2	3	4	5	6	7
h. To exercise at least 30 minutes at a moderate intensity.	1	2	3	4	5	6	7
i. To take time to plan out my meals.	1	2	3	4	5	6	7
j. To try a different physical activity than I usually do or increase the intensity of the activity.	1	2	3	4	5	6	7
k. To modify the way I cook and prepare food (use low-fat substitutes, limit high calorie ingredients, use less salt/sodium, etc.)	1	2	3	4	5	6	7
l. To eat out at restaurants less often than I currently do.	1	2	3	4	5	6	7
m. To make physical activity a priority as much as possible.	1	2	3	4	5	6	7
n. To be physically active even when I don’t feel like it.	1	2	3	4	5	6	7
o. To change my thoughts related to eating and physical activity.	1	2	3	4	5	6	7
p. To weigh myself.	1	2	3	4	5	6	7
q. To wear my pedometer.	1	2	3	4	5	6	7

Feelings and Behaviors

48. **Over the last 2 weeks, how often have you been bothered by any of the following problems:**

	Not at all [0]	Several days [1]	More than half the days [2]	Nearly every day [3]
a. Little interest or pleasure in doing things	☐	☐	☐	☐
b. Feeling down, depressed, or hopeless	☐	☐	☐	☐
c. Trouble falling or staying asleep, or sleeping too much	☐	☐	☐	☐
d. Feeling tired or having little energy	☐	☐	☐	☐
e. Poor appetite or overeating	☐	☐	☐	☐
f. Feeling bad about yourself – or that you are a failure or have let yourself or your family down	☐	☐	☐	☐
g. Trouble concentrating on things, such as reading the newspaper or watching television	☐	☐	☐	☐
h. Moving or speaking so slowly that other people could have noticed. Or the opposite—being so fidgety or restless that you have been moving around a lot more than usual	☐	☐	☐	☐

Medical Forms

Many people have difficulty reading and filling out forms when they go for medical care.

49. How often do you have someone like a family member, hospital worker, clinic worker, or caregiver help you read hospital materials?

- ☐1 Always
- ☐2 Often
- ☐3 Sometimes
- ☐4 Rarely
- ☐5 Never

50. How often do you have problems learning about medical conditions because of difficulty understanding written information?

- ☐1 Always
- ☐2 Often
- ☐3 Sometimes
- ☐4 Rarely
- ☐5 Never

51. How confident are you filling out forms by yourself?

- ☐1 Extremely
- ☐2 Quite a bit
- ☐3 Somewhat
- ☐4 A little
- ☐5 Not at all

52. There was too much reading and writing in the program.

- ☐1 Strongly agree
- ☐2 Agree
- ☐3 Neither agree nor disagree
- ☐4 Disagree
- ☐5 Strongly disagree
- ☐6 Not applicable, I was not in a program

<u>**Group Cohesion**</u>

The following questions are about your experiences with MOVE! or DPP (in-person or online). *If you did not participate in any of the programs, please skip to question 73.*

53. If you had had the chance to switch into a different group working on diet and exercise, how would you have felt about switching?

I would have:

- ☐1 Very much wanted to switch
- ☐2 Rather switched than stayed where I was
- ☐3 Not minded either way
- ☐4 Rather stayed where I was
- ☐5 Very much wanted to stay where I was

54. How comfortable did you feel talking in your group/participating in the online forum?

- ☐1 Very comfortable
- ☐2 Somewhat comfortable
- ☐3 Somewhat uncomfortable
- ☐4 Very uncomfortable

55. How well did you bond with your group members?

- ☐1 I bonded very well with my group members
- ☐2 I bonded with my group members a little
- ☐3 I did not bond with my group members at all

Program Experience and Satisfaction

56. Did you miss any program sessions/online lessons?

- ☐1 No
- ☐2 Yes

57. What was your main reason for not attending or missing sessions/online lessons?

Please choose one answer

- ☐1 Time constraints
- ☐2 Did not have transportation
- ☐3 Financial reasons
- ☐4 Did not think it was important to attend
- ☐5 Sick/not feeling well
- ☐6 Computer or Internet not working properly
- ☐7 Other (please specify): ____
- ☐8 Not applicable, I attended all sessions

58. How did you feel about the number of program sessions/online lessons?

- ☐1 The number of sessions was just right
- ☐2 There were too many sessions
- ☐3 There were too few sessions

59. Did you view any VA DPP sessions on DVD?

- ☐0 Not applicable, I was in MOVE! or the online DPP and did not receive a DVD
- ☐1 No
- ☐2 Yes

a. If **Yes**, how many sessions did you view on DVD?

- ☐1 1
- ☐2 2-3
- ☐3 4-5
- ☐4 6 or more
- ☐5 Unsure

b. If **Yes**, what was the main reason you viewed the DVD?

- ☐1 I missed a VA DPP session
- ☐2 I wanted to review a VA DPP session I attended.
- ☐3 Other (please specify): ___________________________________

60. My group leader motivated me to do my very best.

- ☐1 Strongly agree
- ☐2 Agree
- ☐3 Neither agree nor disagree
- ☐4 Disagree
- ☐5 Strongly disagree

61. When you had important questions to ask your group leader, did you get answers you could understand?

- ☐1 Yes, always
- ☐2 Yes, sometimes
- ☐3 No
- ☐4 Not applicable, I didn’t have questions

62. Did you have confidence and trust in your group leader?

- ☐1 Yes, always
- ☐2 Yes, sometimes
- ☐3 No

63. Did you feel like you were treated with respect and dignity during your group sessions/in the online program?

- ☐1 Yes, always
- ☐2 Yes, sometimes
- ☐3 No

64. Did your group leader provide useful suggestions to help you overcome barriers in meeting your physical activity goals?

- ☐1 Yes, always
- ☐2 Yes, sometimes
- ☐3 No

65. Did your group leader provide meaningful feedback regarding your progress towards meeting your goals?

- ☐1 Yes, always
- ☐2 Yes, sometimes
- ☐3 No

66. Did you find the program hand-outs helpful in changing your dietary intake?

- ☐1 Yes, very helpful
- ☐2 Yes, somewhat helpful
- ☐3 No, not helpful
- ☐4 Not applicable, I was in the online DPP and did not receive handouts

67. Overall, how satisfied are you with the program?

- ☐1 Very satisfied
- ☐2 Satisfied
- ☐3 Neutral
- ☐4 Dissatisfied
- ☐5 Very dissatisfied

68. How likely are you to recommend the program to a friend or family member?

- ☐1 Extremely likely
- ☐2 Likely
- ☐3 Neither likely nor unlikely
- ☐4 Not likely
- ☐5 Would not recommend

69. What did you find to be most helpful about the program?

____

____

70. What was least helpful about the program?

____

____

71. How can we improve the program?

____

____

72. What components of the program should stay the same?

____

____

73. Did you experience any of the health problems listed below related to program activities?

Please check all that apply

- ☐a Muscle or joint injury
- ☐b Dizziness or feeling light headed
- ☐c Falling while exercising
- ☐d High or low blood pressure
- ☐e Heart problems
- ☐f Shortness of breath or breathing difficulties
- ☐g Chest pain
- ☐h Heel pain or plantar fasciitis
- ☐i Foot sores or blisters
- ☐j Dehydration
- ☐k Other (please specify): ___________________________________
- ☐l None

74. What date did you complete this survey?

_____/ _____/ ______

Month Day Year

Thank you for your participation!

Study ID: __ __ __ __ __ __

DIABETES PREVENTION SURVEY

Health Questionnaire

SURVEY COMPLETION DATE:

____ / ____ / ______
Month Day Year

Under each heading, please check the ONE box that best describes your health TODAY

MOBILITY

I have no problems walking ❑

I have slight problems walking ❑

I have moderate problems walking ❑

I have severe problems walking ❑

I am unable to walk ❑

SELF-CARE

I have no problems washing or dressing myself ❑

I have slight problems washing or dressing myself ❑

I have moderate problems washing or dressing myself ❑

I have severe problems washing or dressing myself ❑

I am unable to wash or dress myself ❑

USUAL ACTIVITIES *(e.g. work, study, housework, family or leisure activities)*

I have no problems doing my usual activities ❑

I have slight problems doing my usual activities ❑

I have moderate problems doing my usual activities ❑

I have severe problems doing my usual activities ❑

I am unable to do my usual activities ❑

PAIN / DISCOMFORT

I have no pain or discomfort ❑

I have slight pain or discomfort ❑

I have moderate pain or discomfort ❑

I have severe pain or discomfort ❑

I have extreme pain or discomfort ❑

ANXIETY / DEPRESSION

I am not anxious or depressed ❑

I am slightly anxious or depressed ❑

I am moderately anxious or depressed ❑

I am severely anxious or depressed ❑

I am extremely anxious or depressed ❑

The best health you can imagine

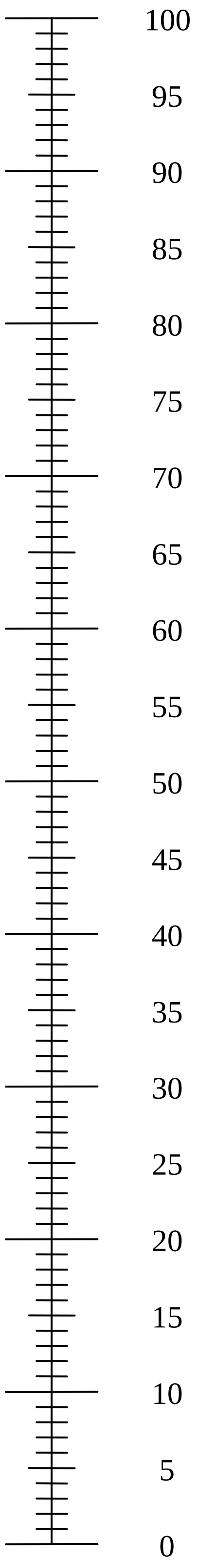

The worst health you can imagine

- We would like to know how good or bad your health is TODAY.
- This scale is numbered from 0 to 100.
- 100 means the <u>best</u> health you can imagine.
  0 means the <u>worst</u> health you can imagine.
- Mark an X on the scale to indicate how your health is TODAY.
- Now, please write the number you marked on the scale in the box below.

YOUR HEALTH TODAY = ☐
