# Supplementary material for: Implementation and evaluation of the VA DPP clinical demonstration: protocol for a multi-site non-randomized hybrid effectiveness-implementation type III trial
Source: Implement Sci. 2015 May 12;10:68. doi: 10.1186/s13012-015-0250-0 (PMC4429938; doi:10.1186/s13012-015-0250-0)
Supplement: Additional file 6: — Patient interview guide. This file provides a copy of the master interview guide used to interview patients. [file 13012_2015_250_MOESM6_ESM.pdf]

VA DPP Study Protocol Additional File 6: Patient Interview guide

Introductions

My name is ________ and I am a researcher working on the project entitled, "VA Diabetes Prevention Program Demonstration Project."

Before we begin, I would like to take a minute to explain why I am inviting you to participate and what I will be doing with the information you provide to me. Please stop me at any time if you have any questions. After I've told you a bit more about my project, you can decide whether or not you would like to participate.

The purpose of this interview is to help us understand your experience with the [VA DPP, VA MOVE! program] and to hear any suggestions you may have about how to improve the program. I am interested in hearing about the things that you especially liked about the program and also any particular challenges you may have encountered during your participation that may have made it hard to meet your goals each week. Your input will help us to evaluate the program and improve it for the future.

Participation is purely voluntary. If you agree to participate in this interview, you will be asked questions related to your experiences in the [VA DPP, VA MOVE! program.] The interview should take approximately 30-60 minutes and will be audio taped so that we do not miss anything that you have to say.

If at any time and for any reason, you would prefer not to answer any questions, please feel free not to answer. If at any time you would like to stop participating, please tell me. We can take a break, stop and continue at a later date, or stop altogether. You will not be penalized in any way for deciding to stop participation at any time. What you say will not affect your participation in the [VA DPP, VA MOVE! program] or your care at the VA. In addition, your group leader is not listening to this nor does s/he have access to any of this data.

I would like to record this interview to make sure that I remember accurately all the information you provide.

Any information you provide will be handled in a confidential manner. Only people working on this study will use the interview recordings. We will take steps to ensure your answers stay confidential.

Your name will not appear on any of the transcripts. Each audio file will be labeled only with a study ID number, which will be linked to the name of the facility in a "crosswalk" file. The crosswalk file will be kept on a secure drive separate from the transcripts. The audio file will be maintained on our secure server. No site or individual names will appear on any of the transcripts. The interview transcript will be given a study ID number, again linked only to the name of the facility, and any personal references that would identify any individuals will be removed.

You will be given a $25.00 gift card as a small token of our appreciation for your assistance with our research project. If you have questions, you are free to ask them now. If you have questions later, you may contact me at 1-800-753-3357.

VA DPP Study Protocol Additional File 6: Patient Interview guide

Are you interested in participating in this study?

If the participant agrees to participate, start the recording and begin the interview. *Interviewers please make a note to probe for the title of any person mentioned by the interviewee but discourage the use of individual names. In addition, please state your name, the participant's ID number, and the date of the interview at the beginning of the recording.*

Construct	Question	Probes
**Introduction:** First, I want to ask you questions about how you became involved with the VA Diabetes Prevention Program/VA MOVE!.		
Enrollment	How did you hear about the VA DPP/VA MOVE!?	
	What made you decide to participate in the program?	• Did you think you needed to make any lifestyle changes?
Prediabetes Diagnosis	Do you have prediabetes?	
	[If yes] Who told you that have prediabetes?	
	[If yes] What does having prediabetes mean to you?	• Did hearing this diagnosis change how you think about your health? Can you tell me more about that?
**General Assessment and Level of Satisfaction**: Now I want to find out how the VA DPP/VA MOVE! is going in general.		
General Assessment	Can you tell me how things have been going for you so far in the VA DPP/VA MOVE!?	
Satisfaction	Are you satisfied with the amount of weight you have lost?	• [If no] Do you plan to continue to lose weight?
	Now I want to ask you a question using a scale. On a scale from 1 to 5, with one being very dissatisfied and 5 being very satisfied, how satisfied are you with:<br><br>The VA DPP/VA MOVE! program in general?	• Why did you rate it this way?
	What did you like about the VA DPP/VA MOVE!?	
	What did you dislike about the VA DPP/VA	

Construct	Question	Probes
	MOVE!?	
	What would you change about the VA DPP/VA MOVE!?	• If this change had been implemented, how would this have affected you?
Participation, Skill Building	If you missed a class lesson, did you feel lost or like you needed to catch up?	• Did it seem like the material built on itself each week?
Satisfaction	How do you feel about how often your group meets and how that changed over time?	• Too often? Not enough?<br>• How did you feel about the increasingly long time between each session?<br>• **VA MOVE! Patients:** Can you tell me about follow-up support you may have received?
**Group Cohesion:** Now I want to talk about your relationships with your group members and your group leader.		
Group Cohesion	Did you have the same group leader(s) during every session?	
	How would you describe your group leader's interaction style with you?	• With the group?<br>• Did you have the opportunity to ask questions?
	What did you think about being in a group setting?	
Group Cohesion, Open/Closed Group	Did the members of your group change during the program?	
Group Cohesion, Group Identity	Did other members of your group have prediabetes?	• Did this affect the group in any way?
Group Cohesion	How would you describe the dynamic within your group?	• Supportive? Competitive?
	How comfortable did you feel talking in your group?	• Was there anything that made it uncomfortable?<br>• Did you ever talk with or correspond with your group leader one-on-one about something you weren't comfortable mentioning in group?
	Have you developed any relationships with the other members outside of the group?	
**Goal Setting:** Now I want to talk to you about goal setting in the VA DPP/VA MOVE!		
Goal Commitment	What would you say the primary goal of the group was in the VA DPP/VA MOVE!?	• To lose weight? Be more physically active? Eat better? Prevent

Construct	Question	Probes
		diabetes?<br>• What did you think about these goals?
	What kind of goals did you have for yourself in the VA DPP/VA MOVE!?	• To what extent were goals assigned for you?<br>○ **DPP Patients**: Do you remember the goal to reduce your body weight by 7% and complete 150 minutes of physical activity each week?<br>• To what extent did you set your own goals?<br>• How did your group leader help you in setting these goals?<br>• Do you feel like you could continue setting goals like this even after the program ends?<br>• **If patient did not set goals:** Why didn't you set goals? Did you take another approach?
Goal Commitment, Skill Building	Did you think your goals were attainable? Why or why not?	• Did it become easier to meet your goals as you attended more sessions?<br>• Did the sessions help you?
Goal Commitment	How much did setting goals help you?	• Can you tell me more about that?<br>• What would have happened if you didn't set goals?
	What were challenges you encountered in achieving your weekly goals for the VA DPP/VA MOVE!?	• What changes did you make to help you meet them?<br>• What are some things that you tried that didn't help?<br>• Are there things that you haven't changed that you think you might need to?<br>• Are there things you are reluctant to change but know you probably should?
	What was the biggest change you made?<br>What was the smallest change you made?	• How did this change over time?<br>• Did the sessions help you do this?
	Did you achieve the goals set or are you on the right track?	• How did this change over time?<br>• Did the sessions help you do this?
Reviewing Progress	Did your group leader review your progress	• Can you tell me more about that?

Construct	Question	Probes
	towards reaching your goal each session?	
	Did your group leader help you brainstorm solutions to challenges you had meeting your goals?	• Can you tell me more about that?
**Self-Monitoring:** Now I want to ask you questions about your food and physical activity diary.		
Self-Monitoring	Did the VA DPP/VA MOVE! increase your awareness of your eating patterns?	• Can you tell me more about that? How did that change over time?<br>• Did the sessions help you do this?
	Did you keep a food diary?	• [If yes] Did you find keeping a diary helpful to keep you on track with your diet goals?<br>• [If yes] Do you still use a food diary? [If no] Why did you stop?<br>• [If no] Why didn't you keep a food diary? Is there anything that could have been done to help you log your food?<br>• How did this change over time?<br>• Did the sessions help you do this?
	Have you changed how you eat?	• Different foods? Different preparation?<br>• How did this change over time?<br>• Did the sessions help you do this?
	What do you think was/is the biggest obstacle to changing how you eat?	• Did this prevent you from reaching your goals?
	How do you feel about your eating since participating in the VA DPP/VA MOVE!?	• Are you satisfied with the amount, frequency, and quality of food you eat?<br>• How often do you feel hungry?<br>• Do you feel like the changes you made are sustainable in the long term?<br>• How often do you eat breakfast?
	Did the VA DPP/VA MOVE! make you think more about your physical activity?	• Can you tell me more about that?
	Did you log your activity?	• [If yes] Did you find logging physical activity helpful to keep you on track with your exercise goals?<br>• [If yes] Do you still log your activity? [If no] Why did you stop?

Construct	Question	Probes
		• [If no] Why didn't you log your physical activity? Is there anything that could have been done to help you log your activity?<br>• How did this change over time?<br>• Did the sessions help you do this?
	What do you think is the biggest obstacle to increasing your physical activity?	• Did this prevent you from reaching your goals?
	Did the VA DPP/VA MOVE! make you feel accountable (i.e., keep you honest) about your diet and physical activity?	• Can you tell me more about that?
**Social Support:** Now I want to talk about how your relationships with your family and friends and how that relates to the VA DPP/VA MOVE!.		
Social Support	Did your participation in the VA DPP/VA MOVE! change your relationships with your family and friends?	• If yes, how?
	Do your family and friends support you in trying to make lifestyle changes?	• If yes, how?
	Did your participation in the VA DPP/VA MOVE! change the activities you do together?	• If yes, how?
	Did you share the VA DPP/VA MOVE! program information with others?	• What did they think?
**Weight-Loss Attempts:** Now I want to talk about previous weight-loss attempts.		
Weight-Loss Attempts	Have you participated in the VA MOVE! before?	
Weight-Loss Attempts, VA DPP/VA MOVE! Comparison	**VA DPP Patients:** If yes, how is the VA DPP different than the VA MOVE!?	• Which program do you prefer?
Weight-Loss Attempts, Comparison	Have you tried other weight loss programs in the past?	• If yes, what was your experience with those programs?<br>• How does it compare with the VA DPP/VA MOVE!?

VA DPP Study Protocol Additional File 6: Patient Interview guide

Construct	Question	Probes
Outside Programs		
**Materials:** Now I want to talk you about the VA DPP/VA MOVE! materials.		
Materials	Do you have suggestions for how we can improve the materials?	• DVDs? Log? Pedometer?<br>• Did you need additional materials? Resistance bands? Exercise DVDs?
**Outcomes and Sustainability:** Now I want to ask you some questions about being able to sustain the changes you have made in the program.		
Outcomes and Sustainability	Did you ever think during the program: "Yes, this is working?"	• Can you tell me more about that?
	How do you picture yourself at this time next year?	
	Do you think the changes you've made will be something you'll be able to continue to do on your own?	**If patient has relapsed:**<br>• Do you think you will be able to make the changes you made in the beginning of the program again? Why or why not?<br>• Can you use the information you have learned in the VA DPP/VA MOVE! to make those changes?
**Closing:** That is all of my questions; is there anything you would like to add? If no, I'd like to thank you for taking the time to share your experiences with us.		
