# Supplementary material for: Implementation and evaluation of the VA DPP clinical demonstration: protocol for a multi-site non-randomized hybrid effectiveness-implementation type III trial
Source: Implement Sci. 2015 May 12;10:68. doi: 10.1186/s13012-015-0250-0 (PMC4429938; doi:10.1186/s13012-015-0250-0)
Supplement: Additional file 7: — Mediation analyses. This file describes the mediation analyses in the “Aim 2” section. [file 13012_2015_250_MOESM7_ESM.pdf]

## VA DPP Study Protocol Additional File 7: Mediation Analyses

The VA DPP evaluation will test whether five program design differences between VA DPP and VA MOVE! impact behavioral constructs and mediate weight loss outcomes between the programs. The hypothesized constructs impacted include goal awareness and commitment, outcome expectations, group cohesion, self-regulation skill mastery, and program intensity. These constructs will be assessed through the baseline and 12-month surveys. Additional File 5 provides a copy of the program and EuroQOL EQ-5D-5L surveys.

To demonstrate mediation, the VA DPP must predict a significant change in the potential mediator, and the VA DPP-induced change in the mediator must explain a significant proportion of variance in weight loss. The linear mixed-effects model examining the intervention effect on weight loss from Aim 2 will be extended for these analyses. The new model will include change from baseline to 12 months for the potential mediator as a covariate, in addition to other baseline covariates (weight, age, and gender, site indicators for the three sites, and a VA DPP or VA MOVE! group indicator). The intervention effect will be compared by adjusting versus not adjusting the model based on the change in the potential mediator. To relax the assumption of no unmeasured confounding for the potential mediator and outcome relationship, mediation analyses based on the structural nested mean model (SNMM) and principal stratification approach will be used [1]. However, as these models require different assumptions, similarity in the conclusions drawn from the different approaches will be reviewed to strengthen confidence in findings. Table 2 in the manuscript details the program design differences and potential behavioral constructs that may mediate weight loss outcomes.

**References**

1. Gallop R, Small DS, Lin JY, Elliott MR, Joffe M, Ten Have TR. Mediation analysis with principal stratification. Statistics in medicine. 2009;28(7):1108-30. doi:10.1002/sim.3533.
