# Supplementary material for: Implementation and evaluation of the VA DPP clinical demonstration: protocol for a multi-site non-randomized hybrid effectiveness-implementation type III trial
Source: Implement Sci. 2015 May 12;10:68. doi: 10.1186/s13012-015-0250-0 (PMC4429938; doi:10.1186/s13012-015-0250-0)
Supplement: Additional file 8: — Cost-effectiveness assessment instructions and log. This file provides a description and instructions for staff to use to log their time. [file 13012_2015_250_MOESM8_ESM.pdf]

VA DPP Study Protocol Additional File 8: Cost-Effectiveness Assessment (CEA) Instructions and Log

CEA Instructions and Log

Purpose: To collect information needed for an economic evaluation of the VA DPP.

Discussion: Separate time into two categories: time spent on the clinical portion of the VA DPP and time spent on the research portion of the VA DPP.

1. Determine staff members that participate in any capacity to implement the VA DPP at each site. These individuals might include: VA DPP staff, VA MOVE! staff (physicians, registered dieticians, and/or schedulers), Computerized Patient Record System (CPRS) staff, and IT staff.

2. Identify activities staff members perform to administer the VA DPP at your site. The goal is to determine what other VA sites would have to do if the VA DPP was implemented at their site. Ask yourself, "Would I do this activity if the VA DPP was offered at my site in the future?" If the answer is "Yes," please log your time as <u>*clinical*</u>. If the answer is "No, I'm doing this for the research portion," then log your time as <u>*research*</u>.

    a. Fill in the amount of time for each activity listed on the CEA Time Log.
    b. If there are additional activities not listed, please list the activity in the blank spaces and enter the time.

    Please note the distance factor and entering patient information is not part of implementing the VA DPP in the future. Therefore, please separate the time of looking up lab values versus time spent checking distance. If this is too difficult, include it as time spent in the clinical portion, as it is better to overestimate than underestimate clinical time.

Instructions for VA DPP Project Coordinators:

1. Give each VA DPP staff member an electronic version of the time log.
2. Ask each staff member to fill out the electronic log daily for one week at three different times during the first year your site enrolls patients into the VA DPP.
    a. The three times logs are to be recorded during: one week in the first month of enrollment, one week during the sixth month of enrollment, and one week during the twelfth month of enrollment. Each site will determine what week during these three time frames staff members should fill out the log. Each staff member should try to fill out the log around the same week.
    b. You may not want to do this task during the week of informed consent since this is a research only activity.

Name:	**VA DPP**<br>**CEA Time Log**				
Role:					
Site:	Hours Spent Doing Each of these Activities				
Date range: _________ to _________	Monday	Tuesday	Wednesday	Thursday	Friday
**Clinical**					
VA DPP eligibility screen (labs, etc)					
A1C/fasting glucose lab order (anything to do with ordering labs for VA DPP eligibility)					
POC lab value - fingerstick (time to do patients during class)					
Scheduling/Reminders VA DPP class (phone calls, letters, CPRS entry, etc)					
VA DPP class prep (gathering class materials, making copies, preparing for class, inventory of materials (handouts, pedos), etc)					
VA DPP class (actual class, CPRS notes, notes to PCPs, etc)					
Administrative tasks specific to VA DPP (returning calls, emails, voicemails, etc)					
Meetings specific to VA DPP					
**Research**					
VA DPP eligibility screen (distance factor ONLY) *Research*					
Data entry for clinical evaluation (patient information, etc) *Research*					

*NOTE: no need to account for time spent on breaks

NOTE: may **not want to do this task during the week of informed consent
